# Effect of Coherent Nanoprecipitate on Strain Hardening of Al Alloys: Breaking through the Strength-Ductility Trade-Off

**DOI:** 10.3390/ma17174197

**Published:** 2024-08-24

**Authors:** Pan Wu, Kexing Song, Feng Liu

**Affiliations:** 1 State Key Laboratory of Solidification Processing, Northwestern Polytechnical University, Xi’an 710072, China; wupan@mail.nwpu.edu.cn; 2 Henan Academy of Sciences, Zhengzhou 450046, China; 3 Analytical & Testing Center, Northwestern Polytechnical University, Xi’an 710072, China

**Keywords:** dislocation density, strain hardening, coherent nanoprecipitate, deformation

## Abstract

So-called strength-ductility trade-off is usually an inevitable scenario in precipitation-strengthened alloys. To address this challenge, high-density coherent nanoprecipitates (CNPs) as a microstructure effectively promote ductility though multiple interactions between CNPs and dislocations (i.e., coherency, order, or Orowan mechanism). Although some strain hardening theories have been reported for individual strengthening, how to increase, artificially and quantitatively, the ductility arising from cooperative strengthening due to the multiple interactions has not been realized. Accordingly, a dislocation-based theoretical framework for strain hardening is constructed in terms of irreversible thermodynamics, where nucleation, gliding, and annihilation arising from dislocations have been integrated, so that the cooperative strengthening can be treated through thermodynamic driving force ∆G and the kinetic energy barrier. Further combined with synchrotron high-energy X-ray diffraction, the current model is verified. Following the modeling, the yield stress $\sigma_{y}$ is proved to be correlated with the modified strengthening mechanism, whereas the necking strain $\varepsilon_{n}$ is shown to depend on the evolving dislocation density and, essentially, the enhanced activation volume. A criterion of high ∆G-high generalized stability is proposed to guarantee the volume fraction of CNPs improving $\sigma_{y}$ and the radius of CNPs accelerating $\varepsilon_{n}$. This strategy of breaking the strength-ductility trade-off phenomena by controlling the cooperative strengthening can be generalized to designing metallic structured materials.

## 1. Introduction

Overcoming the so-called strength–ductility trade-off limitation represents a long-standing and challenging task in physical metallurgy [1,2,3,4,5,6]. The formation of highly dispersed second-phase precipitates with sizes in the nanometer range is an effective phase-engineering strategy for better strength−ductility trade-offs [7,8,9,10]. However, in most cases, precipitation-strengthening increases the strength of the alloy but reduces the ductility [11,12]. The decrease in ductility is mainly attributed to dislocation pileups at the high mismatch interface between reinforcement particles and matrix, which cannot lead to effective dislocation multiplication and annihilation. Nevertheless, recent experiments suggest that through tuning dimensions of coherent nanoprecipitates (CNPs), maraging steels and high-entropy alloys (HEAs) with high strength and good plasticity can be developed [13,14,15,16]. For example, through the introduction of high-density ductile intermetallic L1_2_-type CNPs, the (NiCoFe)_86_Al_7_Ti_7_ alloy exhibits a superior strength of 1500 MPa and a good ductility as high as 50% in tension [14]. The CNPs as a microstructure can effectively promote dislocation multiplication and subsequent dislocation annihilation by restricting the dislocation pileups surrounding the interface, thus leading to the higher ductility [8,9,13]. Nevertheless, the absence of a quantitative resolution among the dimensions of CNPs, the dislocations, and the strength/ductility will make it an enormous challenge to design, artificially and quantitatively, the CNPs for better strength−ductility trade-offs.

Initially, various effective methodologies operating on vastly different time/length scales are interconnected in multi-scale endeavors to describe dislocations [17]. Atomistic techniques at the smallest scales include Density Functional Theory, Molecular Statistics, and Molecular Dynamics [18,19], which address each atom in a system and examine a diverse range of detailed defect physics. Although the simulations have constraints in terms of small-time durations and samples, they offer valuable insights into the active deformation mechanisms, in addition to providing essential data for larger-scale models such as lattice parameters, elastic moduli, kinetic energy barrier, etc. [20]. Advancing to larger scales, there are macro-constitutive approaches that focus on individual defects like dislocations instead of individual atoms. Models at this scale, such as 2D and 3D Discrete Dislocation Dynamics [21,22], Crystal Plasticity [23,24], and Continuum Dislocation Dynamics [25,26], become operative. In most cases, dislocation densities are evolved on separate active slip systems of interest, but can also be resolved in other ways depending on the framework [27]. While this allows for much more realistic samples to be modeled, for example, sizes on the scale of experimental specimens [28], these approaches are still typically too resolved to reach the length scales needed to design and model components since the individual slip systems and microstructure, such as the CNPs, are explicitly resolved.

Lately, this discussion focuses on models that operate on the largest length and time scales, which analyze the plastic strain or mobile, immobile, or total dislocation densities. Several methods are based on the framework created by Kocks and Mecking (K-M), involving the evolution of total dislocation density and considering dislocation storage and dynamic recovery [29]. As for precipitates, a semi-empirical model has been established to comprehend the strain hardening rate with various precipitate distributions [30]. Nevertheless, the essential fitting parameters associated with precipitation seem to be inadequate for tuning the dimensions of precipitates for target properties. On this basis, the classical K-M equation was modified by adding a coupled term, which accounts for volume fraction $V_{f}$ and radius R of the precipitates affecting Orowan loops [31]. In addition, to take into account the morphology of precipitates [32], the contribution from plastic relaxation around the precipitate plates that accompanies the internal stress saturation to strain hardening is quantitatively established by coupling the length of shear-resistant plate-shaped precipitate [5]. Further, for the thermally activated process, the kinetic energy barriers of dislocation bend and kink-pair formation are quantified using precipitate diameter and spacing [33]. However, owing to individual strengthening of the Orowan or the shearing mechanism, this model is not appropriate for the CNPs-strengthened alloys that are strengthened by cooperative mechanisms [34].

Simultaneously promoting the yield stress $\sigma_{y}$ and the uniform elongation $\varepsilon_{n}$ of the CNPs-strengthened alloys require detailed knowledge of plastic deformations (PDs), which can be uniformly described as the kinetic behaviors of atoms triggered by thermodynamic driving force ∆G [35], including nucleation, gliding, and annihilation arising from dislocations [34,36,37,38]. As for the dislocation nucleation, generally, a critical stress $\sigma_{y}$ is defined as the point that breaks the mechanical stability, which corresponds to the loss of thermodynamic stability [39]. As for the dislocation gliding and annihilation, except from a dependence on Taylor factor, the necking onset strain or the uniform elongation must be governed by the dislocation recovery kinetics [40,41], as reflected by the gliding velocity, $v=v_{0}exp\left(-Q/k_{B}T\right)$ [42], with $v_{0}$ as the atomic vibration frequency, $k_{B}$ the Boltzmann’s constant, T the absolute temperature, and Q the kinetic energy barrier [43]. Following so-called thermo-kinetic correlation prevailing upon PDs [35,37,44], the lower applied shear stress τ, physically and mathematically, corresponds to the higher Q [44,45], thus giving the lower gliding velocity, and in turn, the weaker dislocation recovery or the higher $\varepsilon_{n}$ [40,41]. In this regard, the strength–ductility trade-off phenomenon physically reflects the thermo-kinetic trade-off relationship between τ and Q for dislocation [44,45]. From a thermo-kinetic perspective, how to suppress the dislocation gliding subjected to the high flow stress identically corresponds to the high τ and high Q for dislocation gliding [35,46].

Based on the irreversible thermodynamic framework considering nucleation, gliding, and annihilation arising from dislocations [42,47], this work aims to develop a dislocation-based strain hardening model, where the cooperative strengthening of multiple interactions between CNPs and dislocations can be considered using dislocation thermo-kinetics as a bridge. Taking Al-Mg-Si alloy as an example, the dislocation density was measured using the synchrotron high-energy X-ray diffraction (HEXRD) technique, as illustrated in Section 2. Considering nucleation, gliding, and annihilation arising from dislocations, Section 3 deduced the dislocation-based strain hardening model. The yield strength increment, the stress-strain curves, and the dislocation density calculated by the current model are demonstrated in Section 4. Model prediction in Section 5 quantitatively established the mechanisms of $\sigma_{y}$ and $\varepsilon_{n}$ regulated by the $V_{f}$ and the R of CNPs. Further integrating the GS with dislocation thermo-kinetics, the trade-offs between $\sigma_{y}$ and $\varepsilon_{n}$, and the breaking of trade-offs are analyzed in Section 6. On this basis, a new strategy is proposed: high ∆G for dislocations nucleation is controlled by the $V_{f}$ of CNPs improving $\sigma_{y}$, and high GS for dislocations gliding is controlled by the appropriate R of CNPs accelerating $\varepsilon_{n}$. Finally, the main conclusions are summarized in Section 7. The symbols used in the paper are defined where they first appear; for convenience, they are also assembled with their definitions in Table 1.

## 2. Materials and Methods

This experiment used 5-mm-thick cold-rolled plates of Al-Mg-Si alloy manufactured by twin-roll casting with the chemical composition shown in Table 2. The cold-rolled plates were cut into disk samples of 10 mm in diameter and 1.5 mm in thickness using wire cutting. The disk samples underwent solution heat treatment at 540 °C for 1 h and were water-quenched to room temperature. After water quenching, the samples were naturally aged at different times to form the CNPs.

Spherical aberration corrected transmission electron microscopy (ACTEM) (FEI, Hillsboro, O R, USA) was employed to examine the CNPs, where, the ACTEM specimens were prepared using jet polishing with a solution of 30% nitric acid in methanol below −25 °C at an operating voltage of 15 V. Tensile samples of 32 mm in length, 6 mm in width, and 1.2 mm in thickness were cut from the sheet along the rolling orientation and tested at a nominal strain rate of 1 × 10^−4^ s^−1^ at room temperature. Four tensile specimens were examined for each condition to confirm the test findings’ reproducibility.

Evolution of dislocation density was measured using the HEXRD experiments conducted on tensile specimens at Beam Line No. BL14B1 of Shanghai Synchrotron Radiation Facility (SSRF) (Shanghai, China). The energy of the monochromatic synchrotron X-ray beam was 18 keV, which corresponds to a wavelength of 0.688700 nm. LaB6 powder was used for calibrating the instrumental profile. Two-dimensional (2-D) diffraction patterns were captured in reflection mode using an image plate detector and then integrated into one-dimensional (1-D) line profiles using the FIT2D software package (Version: V12.077). The dislocation densities of tensile specimens were calculated using a combination of the modified Williamson–Hall (MWH) method and the modified Warren–Averbach (MWA) method [48].

## 3. Dislocation-Based Strain Hardening Model Framework

### 3.1. Philosophy, Framework, and Assumptions

**Figure 1:**
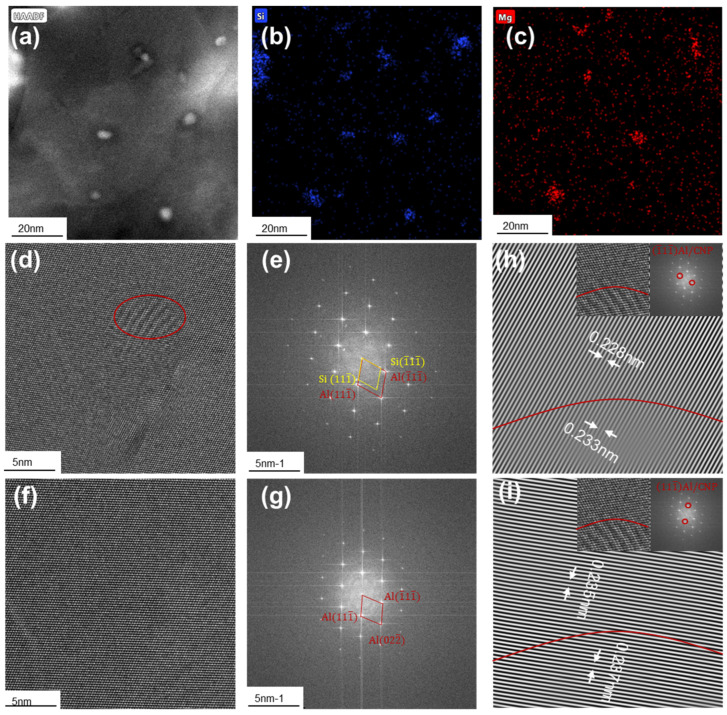
ACTEM images of the coherent nanoprecipitates (CNPs) obtained from Al-Mg-Si samples. (**a**) High-angle annular dark field (HAADF) image, (**b**,**c**) corresponding EDX mappings of Si and Mg components. (**d**) High Resolution Transmission Electron Microscope (HRTEM) and (**e**) corresponding Fast Fourier transform (FFT) images, where CNP and α-Al are divided by the red circle. (**f**) HRTEM and (**g**) corresponding FFT images showing α-Al matrix. (**h**,**i**) CNP and their corresponding IFFT images. The zone axis is [110] for α-Al and CNP.

Before irreversible PDs, the yield stress increment produced by the precipitates is dictated by so-called resistive force *K* of precipitates acting on the dislocations at thermodynamic equilibrium, where *K* is balanced by the line tension *Γ* of bowed dislocation segments [49,50]. Once the thermodynamic equilibrium is disturbed, it is theoretically feasible to overcome the plastic resistance with thermal assistance at a stress lower than the shear resistance [51,52]. In this case, the thermal-activated kinetic energy barrier Q also relies on the K [51], which varies in response to typical interactions between precipitates and dislocations [49]. ACTEM is used to characterize the interactions between CNPs in Al-Mg-Si alloys and dislocations using an electron beam-aligned parallel to [001]_Al_ zone axis. The CNP are enriched in Si and Mg components and have a diameter of several nanometers (Figure 1a–c); such nanoscale particles have been acknowledged in Refs. [53,54,55]. A High Resolution Transmission Electron Microscope (HRTEM) image (Figure 1d) shows CNP and α-Al matrix separated by a red circle, as identified by the Fast Fourier transform (FFT) pattern (Figure 1e). The FFT pattern from the [001]_Al_ exhibits a typical face-centered cubic (fcc) structure of the α-Al matrix, with weak spots identified as [001]_CNP_ from an ordered structured phase. Figure 1f depicts an HRTEM image of the α-Al matrix, distinguished by the associated FFT pattern (Figure 1g). In contrast to Figure 1e, the FFT pattern displays the characteristic fcc structure without additional super-lattice diffraction spots. First, the orientation relations between CNP and α-Al are [110]_Al_//[110]_CNP_, ($\bar{1}1\bar{1}$)_Al_//($\bar{1}1\bar{1}$)_CNP_, and ($11\bar{1}$)_Al_//($11\bar{1}$)_CNP_. Second, the interplanar spacings for ($\bar{1}1\bar{1}$) of CNP and α-Al are 0.233 and 0.228 nm, respectively, indicating a lattice mismatch of 0.02 (< 0.05); and the interplanar spacings for ($11\bar{1}$) of CNP and α-Al are 0.237 and 0.235 nm, respectively, indicating a lattice mismatch of 0.008 (<0.05). Third, no misfit dislocations are observed at the interface of CNP and α-Al. Hence, the CNP are ordered and coherent with the α-Al matrix. As such, precipitate shearing, precipitate bypass by Orowan looping, or a combination of these two mechanisms account for ambient-temperature strength in precipitation-strengthened alloys ([49,53], see also Figure 1h,i). With references to [34,37,49], the dominated mechanism depends on the relative magnitude of Γ and the peak resistive force $\hat{K}$. As for $\hat{K}\leq 2\Gamma$, the CNPs can be shared by the dislocation, thus forming two contributions [56,57,58]: (i) coherency strengthening, i.e., dislocation is hindered by the coherent strain generated by lattice distortion between the CNPs and the matrix [59]; and (ii) order strengthening, i.e., dislocation disorders the CNPs upon shearing and produces the antiphase boundaries (APB) [49]. As for $\hat{K}>2\Gamma$, the CNPs can no longer be sheared but will be bypassed as the dislocation bows around such shear-resistant precipitates, pinches off, and leaves behind loops [37].

**Figure 2:**
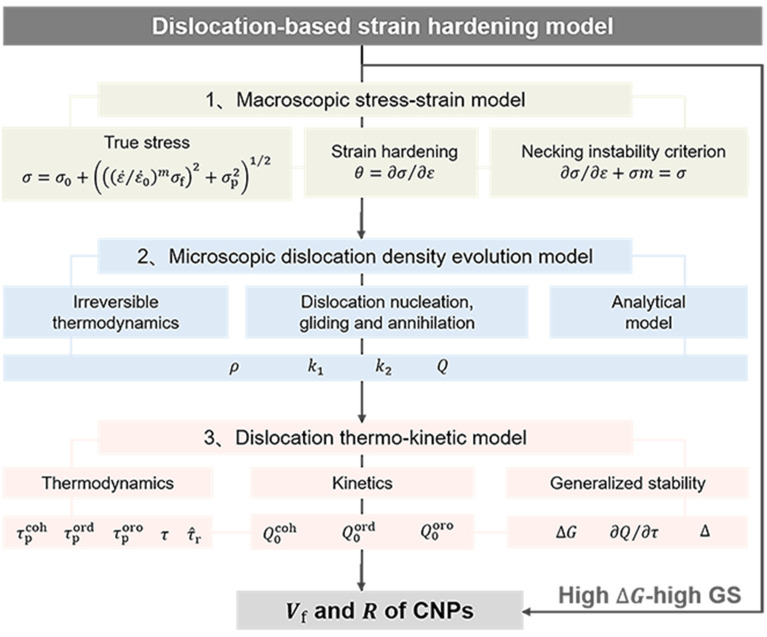
Modular framework for the present dislocation-based strain hardening model of CNP-strengthened alloys.

Following the above philosophy, a concise framework for the dislocation-based strain hardening model is shown in Figure 2. Firstly, the true stress, the strain hardening, and the necking strain are described by establishing a macroscopic stress-strain model, where the true stress is generally calculated as [5,30]: $\sigma =\sigma_{0}+{\left({\left({\left(\dot{\varepsilon }/{\dot{\varepsilon }}_{0}\right)}^{m}\sigma_{f}\right)}^{2}+\sigma_{p}^{2}\right)}^{1/2}$, with $\sigma_{0}$ as the lattice stress, $\sigma_{f}$ the forest dislocation stress, $\sigma_{p}$ the precipitate stress, and m the strain rate sensitivity exponent; the strain hardening rate ∂σ/∂ε is calculated by numerical differentiation, and the necking strain is determined by the plastic instability condition: ∂σ/∂ε+σm=σ [40,60]. In this paper, as the Al-Mg-Si alloy is a typical single–principal-element alloy (following the traditional theory [61,62,63,64], as R increases, $\sigma_{p}$ is determined by the mechanism with the lower value between due to the shearing and due to the Orowan mechanism. However, the prevalent mechanism due to the shearing mechanism becomes controversial if subjected to the coherency and/or the order strengthening as follows. One theory believes that the main strengthening is determined by the highest value due to the order or the coherency strengthening [63,64]; the maximum effect due to the coherent strengthening comes once the shearing dislocation is near the CNPs interface, whereas the maximum effect is due to the order strengthening by shearing half the CNPs. Another idea suggests that the major strengthening comes from the lowest value of the order or the coherency strengthening [61,62]; for single-principal-element alloys, strengthening is nearly independent of R, as predicted by order strengthening [58,63]), the term $\sigma_{p}$ can be analogous to Refs. [61,62,63,64] expressed with a simple but rather artificial method: $\sigma_{p}=min\left\{\sigma_{p}^{coh},\sigma_{p}^{oro},\sigma_{p}^{ord}\right\}$, with $\sigma_{p}^{coh}$, $\sigma_{p}^{ord}$ and $\sigma_{p}^{oro}$ respectively as the stress due to coherency, order and Orowan strengthening; see Equations (S1)–(S4) in the Supplementary Materials. Secondly, based on the irreversible thermodynamics, a microscopic dislocation model is constructed by coupling nucleation, gliding, and annihilation arising from dislocations, where the Q for cooperative strengthening of coherency, order, or Orowan mechanism is deduced analytically. Thirdly, a thermo-kinetic model for dislocation evolution is established to model ∆G and Q for individual strengthening using $V_{f}$ and R of the CNPs, so that a high ∆G-high GS criterion can be proposed to improve the strength and ductility, simultaneously.

For simplicity, several assumptions are provided as follows: (1) the kinematic hardening (long-range internal stress opposing plastic straining) produced by the CNPs is neglected due to the weak influence of kinematic hardening on $\varepsilon_{n}$ [5]; (2) only the spherical CNPs are considered, as seen in Figure 1; and (3) the effect of grain size on the evolution of dislocation density is neglected due to its weak influence on CNPs strengthened alloys in this study [65].

### 3.2. A Model for Dislocation Density

#### 3.2.1. A Concise Description for Irreversible Thermodynamics of Dislocation

Analogous to the concept of equilibrium state, a stationary non-equilibrium state can be defined in the context of irreversible thermodynamics, which reduces the entropy generation rate ($d_{i}S/dt$) under certain external constraints. The total entropy generation can be expressed as [42]:

(1) $$\frac{dS}{dt}=\frac{d_{e}S}{dt}+\frac{d_{i}S}{dt}$$

with the term dS/dt as the total entropy change rate and the term $d_{e}S/dt$ the entropy flux rate between the system and the surroundings.

There are usually three irreversible processes related to dislocations producing entropy $d_{i}S$: nucleation, gliding, and annihilation [42,47]. Within a shear strain interval dγ, the $d\rho^{+}$ dislocations are generated at energetically advantageous regions by Frank–Read sources. The generated dislocations subsequently glide through the CNPs to eventually form gliding bands or Orowan loops [62]. Meanwhile, the $d\rho^{-}$ dislocations are annihilated by various mechanisms, including kinetic recovery. These three activities are irreversible processes which, according to the theory of irreversible thermodynamics, produce entropy $d_{i}S$ [42,47]:

(2) $$d_{i}S=\frac{dW_{ge}}{T}+\frac{dW_{gl}}{T}+\frac{dW_{an}}{T}$$

with $dW_{ge}$, $dW_{gl}$ and $dW_{an}$ as the dissipated energies due, respectively, to the process of generation, gliding, and annihilation of dislocation. Note that the $dW_{ge}$ is assumed to be proportional to the $d\rho^{+}$ [42]:

(3) $$dW_{ge}=Ed\rho^{+}$$

with $E=G_{m}b^{2}/2$ as the elastic energy of dislocations per unit length, $G_{m}$ the shear modulus of matrix, and b the magnitude of Burgers vector. The energy dissipated due to the dislocation gliding, $dW_{gl}$, is expressed as [42]:

(4) $$dW_{gl}=\tau_{r}bl^{s}d\rho^{+}$$

with $l^{s}=1/\sqrt{\rho }$ as the mean gliding distance and $\tau_{r}$ the average shear resistance.

As dislocations are annihilated, their elastic energies will be dissipated and released into the surroundings. Therefore, the $dW_{an}$ is expressed as [42]:

(5) $$dW_{an}=Ed\rho^{-}$$

with $d\rho^{-}$ as the annihilation of dislocation recovery. Combining Equations (3)–(5), Equation (2) can be rewritten as:

(6) $$d_{i}S=\left(1+\frac{2\tau_{r}}{G_{m}b\sqrt{\rho }}\right)\frac{G_{m}b^{2}}{2T}d\rho^{+}+\frac{G_{m}b^{2}}{2T}d\rho^{-}$$

At the unsteady state, the difference between dislocation generation and annihilation is determined as the average dislocation density ρ, which gives:

(7) $$d\rho =d\rho^{+}-d\rho^{-}$$

Substituting Equation (7) into Equation (8), $d_{i}S$ is rewritten as [42]:

(8) $$d_{i}S=\left(1+\frac{2\tau_{r}}{G_{m}b\sqrt{\rho }}\right)\frac{G_{m}b^{2}}{2T}d\rho +\left(2+\frac{2\tau_{r}}{G_{m}b\sqrt{\rho }}\right)\frac{G_{m}b^{2}}{2T}d\rho^{-}$$

The entropy flux $d_{e}S$ for a shear strain increment dγ is related to the heat flux between the deformed metal and the environment [42]:

(9) $$d_{e}S=\frac{dU-dW}{T}$$

with $dU=1/2G_{m}b^{2}d\rho$ as the dislocations storage energy in the material as PD takes place, $dW=\tau_{f}d\gamma$ as the mechanical work done into the metal, and $\tau_{f}$ as the shear stress of forest dislocation. On addition of $d_{i}S$ and $d_{e}S$, the total entropy change then becomes [42]:

(10) $$\frac{dS}{d\gamma }=\left(2+\frac{2\tau_{r}}{G_{m}b\sqrt{\rho }}\right)\frac{G_{m}b^{2}}{2T}\frac{d\rho }{d\gamma }+\left(2+\frac{2\tau_{r}}{G_{m}b\sqrt{\rho }}\right)\frac{G_{m}b^{2}}{2T}\frac{d\rho^{-}}{d\gamma }-\frac{\tau_{f}}{T}$$

Equation (10) establishes a connection between entropy and PDs via the development of dislocation structure. Since both the strain hardening rate and the entropy change vanish at the steady state ($d\tau_{f}/d\gamma =dS/d\gamma$ = 0), it is hypothesized that [42,66]:

(11) $$\frac{dS}{d\gamma }=\frac{C}{T}b\sqrt{\rho }\frac{d\tau_{f}}{d\gamma }$$

with C as the temperature-dependent constant and $b\sqrt{\rho }$ the scaling parameter. Note that at the steady state, both the left- and right-hand sides of this equation become zero. The strain hardening rate can be expressed as [42]:

(12) $$\frac{d\tau_{f}}{d\gamma }=\frac{\alpha G_{m}b}{2\sqrt{\rho }}\frac{d\rho }{d\gamma }$$

Integrating Equations (10)–(12) offers [42]:

(13) $$\frac{d\rho }{d\gamma }=\frac{\alpha }{b}{\left(1+\tau_{r}\frac{1}{G_{m}b\sqrt{\rho }}-\frac{C\alpha }{2}\right)}^{-1}\sqrt{\rho }-\left(1+\frac{\tau_{r}}{G_{m}b\sqrt{\rho }}\right){\left(1+\tau_{r}\frac{1}{G_{m}b\sqrt{\rho }}-\frac{C\alpha }{2}\right)}^{-1}\frac{d\rho^{-}}{d\gamma }$$

#### 3.2.2. Further Thermo-Kinetic Introduction in Present Modeling

Departing from Equation (13), it is herein proposed to consider the average shear resistance $\tau_{r}$ for the dislocation gliding, which is expressed as [5]:

(14) $$\tau_{r}=\tau_{0}+{\left(\tau_{p}^{2}+\tau_{fr}^{2}\right)}^{1/2}$$

with $\tau_{0}$ as the shear resistance of lattice, $\tau_{fr}=\alpha^{’}G_{m}b\sqrt{\rho_{m}}$ as the shear resistance of forest dislocation, with $\alpha^{’}$ as the geometrical factor that depends on type and arrangement of the interacting dislocations, and $\rho_{m}$ as the mobile dislocation density, which is expected to increase with the average dislocation density ρ upon PDs. In particular, the ratio of $\tau_{fr}$ and the shear stress of forest dislocation $\tau_{f}=\alpha G_{m}b\sqrt{\rho }$ holds almost constant [34,37,42,46], with α as the strengthening coefficient varying within a wide range of 0.1∼0.5. Fortunately, the average shear resistance $\tau_{r}$ for dislocation is quantified, aiding in the quantification of effective driving force of dislocation (see Section 3.3).

Further following Kocks and Mecking, the annihilation of dislocations in fcc metals can be expressed quantitatively as [42]:

(15) $$\frac{d\rho^{-}}{d\gamma }=\frac{Nv}{\dot{\gamma }}\rho =\frac{{Nv}_{0}}{\dot{\gamma }}exp\left(-\frac{Q}{k_{B}T}\right)\rho$$

with v as the velocity of dislocations gliding, N the number of dislocation jogs per unit length, $v_{0}$ the atomic vibration frequency, γ the shear strain, and $\dot{\gamma }$ the shear strain rate. When examining the annihilation of dislocation via the obstruction of CNPs, as outlined in Section 3.1, three forms of hindrance exist: strain energy, APB, and dislocation bending. According to Refs. [61,62], the total yield increment is determined by selecting the minimum value among the coherency, the order, and the Orowan strengthening. Therefore, the dislocation annihilation induced by three individual strengthening effects can be considered as additive [43], and thus expressed as:

(16) $$\frac{d\rho^{-}}{d\gamma }=\frac{N^{ord}v_{0}}{\dot{\gamma }}exp\left(-\frac{Q_{0}^{ord}}{k_{B}T}\left(1-\frac{\tau }{{\hat{\tau }}_{r}^{ord}}\right)\right)\rho +\frac{N^{coh}v_{0}}{\dot{\gamma }}exp\left(-\frac{Q_{0}^{coh}}{k_{B}T}\left(1-\frac{\tau }{{\hat{\tau }}_{r}^{coh}}\right)\right)\rho \;+\frac{N^{oro}v_{0}}{\dot{\gamma }}exp\left(-\frac{Q_{0}^{oro}}{k_{B}T}\left(1-\frac{\tau }{{\hat{\tau }}_{r}^{oro}}\right)\right)\rho$$

with $N^{ord}$, $N^{coh},$ and $N^{oro}$ as the number of CNPs, respectively, for the order, the coherency, and the Orowan strengthening, and can be calculated by [9]: $N^{x}={\left(V_{f}\right)}^{x}/\left(\left(4/3\right)\pi {\left(R^{x}\right)}^{3}\right),\;x=coh,ordandoro$. The terms $Q_{0}^{ord}$, $Q_{0}^{coh},$ and $Q_{0}^{oro}$ represent the zero-stress energy barrier, whereas ${\hat{\tau }}_{r}^{ord}$, ${\hat{\tau }}_{r}^{coh},$ and ${\hat{\tau }}_{r}^{oro}$ denote the peak shear resistance related to the order, the coherency, and the Orowan strengthening, respectively. These are defined in Equations (S5)–(S7) in the Supplementary Materials. The first and the second terms in the right-hand side of Equation (16) can be redescribed as:

(17) $$\frac{d\rho_{s}^{-}}{d\gamma }=\frac{v_{0}}{\dot{\gamma }}\rho \left(N^{coh}exp\left(-\frac{Q_{0}^{coh}}{k_{B}T}\left(1-\frac{\tau }{{\hat{\tau }}_{r}^{coh}}\right)\right)+N^{ord}exp\left(-\frac{Q_{0}^{ord}}{k_{B}T}\left(1-\frac{\tau }{{\hat{\tau }}_{r}^{ord}}\right)\right)\right)$$

Equation (17) describes the dislocation annihilation rate including two sub-processes, i.e., coherency and order strengthening. In Equation (17), the kinetic parameters $N^{coh}$, $N^{ord}$, $Q_{0}^{coh},$ and $Q_{0}^{ord}$ are independent functions of dislocation density ρ. This provides further evidence for the validity of the additivity rule for dislocation density. The additivity provides credibility for using analytical models to deal with the influence of cooperative effects of various kinetic processes on dislocation annihilation [67]. Analogous to Refs. [68,69,70], two positive integers $r_{1}$ and $r_{2}$ are introduced to characterize the relative contributions ratio of the above two sub-processes. Hence, the complete dislocation annihilation is divided into $r_{1}+r_{2}$ components, and the further derivation gives Equation (17) as:

$$\frac{d\rho_{s}^{-}}{d\gamma }=\frac{v_{0}\rho }{\left(r_{1}+r_{2}\right)\dot{\gamma }}$$

(18) $$\left(\begin{array}{c} N^{coh}\left(1+r_{1,2}\right)exp\left(-\frac{Q_{0}^{coh}}{k_{B}T}\left(1-\frac{\tau }{{\hat{\tau }}_{r}^{coh}}\right)\right)+N^{ord}(1+r_{1,2}^{-1})exp\left(-\frac{Q_{0}^{ord}}{k_{B}T}\left(1-\frac{\tau }{{\hat{\tau }}_{r}^{ord}}\right)\right) \end{array}\right)$$

where

(19) $$r_{1,2}=\frac{r_{2}}{r_{1}}=\left(N^{ord}exp\left(-\frac{Q_{0}^{ord}}{k_{B}T}\left(1-\frac{\tau }{{\hat{\tau }}_{r}^{ord}}\right)\right)\right)/\left(N^{coh}exp\left(-\frac{Q_{0}^{coh}}{k_{B}T}\left(1-\frac{\tau }{{\hat{\tau }}_{r}^{coh}}\right)\right)\right)$$

Based on the analogous summation/product transition [68,69,70], since each component inside the sum in brackets is identical, Equation (18) can be further reformulated as a product:

$$\frac{d\rho_{s}^{-}}{d\gamma }=\frac{v_{0}\rho }{\dot{\gamma }}\left({N^{coh}\left(1+r_{1,2}\right)}^{\frac{1}{1+r_{1,2}}}+N^{ord}{\left(1+r_{1,2}^{-1}\right)}^{\frac{1}{1+r_{1,2}^{-1}}}\right)$$

(20) $$exp\left(-\frac{1}{r_{1}+r_{2}}\left(\frac{r_{1}Q_{0}^{coh}}{k_{B}T}\left(1-\frac{\tau }{{\hat{\tau }}_{r}^{coh}}\right)+\frac{r_{2}Q_{0}^{ord}}{k_{B}T}\left(1-\frac{\tau }{{\hat{\tau }}_{r}^{ord}}\right)\right)\right)$$

Following the approach in Equations (17)–(20) and combining Equation (20) and the third component in the right-hand of Equation (16), i.e., the dislocations annihilated caused by the dislocation bending, the $d\rho^{-}/d\gamma$ can be further derived as:

$$\frac{d\rho^{-}}{d\gamma }=\frac{v_{0}\rho }{\dot{\gamma }}{\left(N^{oro}\left(1+r_{3,4}^{-1}\right)\right)}^{\frac{1}{1+r_{3,4}^{-1}}}$$

$${\left(\left(N^{coh}{\left(1+r_{1,2}\right)}^{\frac{1}{1+r_{1,2}}}+{N^{ord}\left(1+r_{1,2}^{-1}\right)}^{\frac{1}{1+r_{1,2}^{-1}}}\right)\left(1+r_{3,4}\right)\right)}^{\frac{1}{1+r_{3,4}}}$$

(21) $$exp\left(-\frac{1}{r_{1}+r_{2}+r_{3}}\left(\frac{r_{1}Q_{0}^{coh}}{k_{B}T}\left(1-\frac{\tau }{{\hat{\tau }}_{r}^{coh}}\right)+\frac{r_{2}Q_{0}^{ord}}{k_{B}T}\left(1-\frac{\tau }{{\hat{\tau }}_{r}^{ord}}\right)+\frac{r_{3}Q_{0}^{oro}}{k_{B}T}\left(1-\frac{\tau }{{\hat{\tau }}_{r}^{oro}}\right)\right)\right)$$

where

(22) $$r_{3,4}=\frac{r_{3}}{r_{1}+r_{2}}=\frac{N^{oro}exp\left(-\frac{Q_{0}^{oro}}{k_{B}T}\left(1-\frac{\tau }{{\hat{\tau }}_{r}^{oro}}\right)\right)}{N^{coh}exp\left(-\frac{Q_{0}^{coh}}{k_{B}T}\left(1-\frac{\tau }{{\hat{\tau }}_{r}^{coh}}\right)\right)+N^{ord}exp\left(-\frac{Q_{0}^{ord}}{k_{B}T}\left(1-\frac{\tau }{{\hat{\tau }}_{r}^{ord}}\right)\right)}$$

Integrating Equations (14) and (22), the total dislocation density evolution can be expressed as:

(23) $$\frac{d\rho }{d\gamma }=k_{1}\sqrt{\rho }-k_{2}\rho$$

where

(24) $$k_{1}=\frac{\alpha }{b}{\left(1+\frac{\tau_{r}}{G_{m}b\sqrt{\rho }}-\frac{C\alpha }{2}\right)}^{-1}$$

$$k_{2}={\frac{v_{0}}{\dot{\gamma }}\left(1+\tau_{r}\frac{1}{G_{m}b\sqrt{\rho }}\right)\left(1+\tau_{r}\frac{1}{G_{m}b\sqrt{\rho }}-\frac{C\alpha }{2}\right)}^{-1}{\left(N^{oro}\left(1+r_{3,4}^{-1}\right)\right)}^{\frac{1}{1+r_{3,4}^{-1}}}$$

$${\left(\left(N^{coh}{\left(1+r_{1,2}\right)}^{\frac{1}{1+r_{1,2}}}+{N^{ord}\left(1+r_{1,2}^{-1}\right)}^{\frac{1}{1+r_{1,2}^{-1}}}\right)\left(1+r_{3,4}\right)\right)}^{\frac{1}{1+r_{3,4}}}$$

(25) $$exp\left(-\frac{1}{r_{1}+r_{2}+r_{3}}\left(\frac{r_{1}Q_{0}^{coh}}{k_{B}T}\left(1-\frac{\tau }{{\hat{\tau }}_{r}^{coh}}\right)+\frac{r_{2}Q_{0}^{ord}}{k_{B}T}\left(1-\frac{\tau }{{\hat{\tau }}_{r}^{ord}}\right)+\frac{r_{3}Q_{0}^{oro}}{k_{B}T}\left(1-\frac{\tau }{{\hat{\tau }}_{r}^{oro}}\right)\right)\right)$$

Compared with the standard K-M equation, the dislocation storage coefficients $k_{1}$ and the kinetic recovery coefficients $k_{2}$ in this work are mathematically correlated with the thermodynamic parameters and the kinetic parameters. Equations (14) and (24) indicate that there are three kinds of contributions to dislocation storage, i.e., forest dislocation storage, lattice resistance storage, and CNP storage. Equation (25) reflects three types of kinetic energy barriers for dislocation gliding formed by the APB, the strain energy, and the dislocation bending. The thermodynamic and the kinetic parameters will be further quantitatively correlated with the R and the $V_{f}$ of CNPs in Section 3.3. Based on this, it is feasible to predict the dislocation density, the yielding strength, and the necking strain by altering R and $V_{f}$ of the CNPs.

### 3.3. A Thermo-Kinetic Model for Dislocation Evolution

As the dislocations interact with the CNPs, the alloys are strengthened since the CNPs generate the thermodynamic resistance to the dislocations [34,37]. If the friction resistance, the precipitate resistance, and the forest dislocation resistance are all considered, the thermodynamic driving force for the dislocation gliding ∆G is defined by the difference between driving force from applied stress ($∆G_{\tau }$) and gliding resistance ($∆G_{\tau_{r}}$) [34,37,45,71]:

(26) $$∆G=∆G_{\tau }-∆G_{\tau_{r}}=\tau -\left(\tau_{0}+{\left(\tau_{fr}^{2}+\tau_{p}^{2}\right)}^{1/2}\right)$$

According to empirical power law [72], the applied shear stress τ can be expressed as: $\tau =\tau_{0}+{\left({\left({\left(\dot{\gamma }/{\dot{\gamma }}_{0}\right)}^{m}\tau_{f}\right)}^{2}+\tau_{p}^{2}\right)}^{1/2}$, with ${\dot{\gamma }}_{0}$ as the reference strain rate. The relationship between m and $V^{*}$ is described by $m=k_{B}T/V^{*}\tau$. At the yielding point, $\tau_{f0}=\alpha G_{m}b\sqrt{\rho_{0}}$ holds, with $\rho_{0}$ as the initial dislocation density. Thus, the applied shear stress at the yielding point can be expressed as: $\tau_{y}=\tau_{0}+{\left({\left({\left(\dot{\gamma }/{\dot{\gamma }}_{0}\right)}^{m}\tau_{f0}\right)}^{2}+\tau_{p}^{2}\right)}^{1/2}$.

According to Equation (25), the Q assuming cooperative strengthening of order, coherency, and Orowan strengthening mechanisms is:

(27) $$Q=\left(Q_{0}-\frac{\tau }{r_{1}+r_{2}+r_{3}}\left(\frac{r_{1}Q_{0}^{coh}}{{\hat{\tau }}_{r}^{coh}}+\frac{r_{2}Q_{0}^{ord}}{{\hat{\tau }}_{r}^{ord}}+\frac{r_{3}Q_{0}^{oro}}{{\hat{\tau }}_{r}^{oro}}\right)\right)$$

where

(28) $$Q_{0}=\left(r_{1}Q_{0}^{coh}+r_{2}Q_{0}^{ord}+r_{3}Q_{0}^{oro}\right)/\left(r_{1}+r_{2}+r_{3}\right)$$

Substituting Equation (26) into Equation (27), then it is obtained,

$$\left(r_{1}+r_{2}+r_{3}\right)\left(Q-Q_{y}\right)=-\left(∆G+\tau_{0}+\tau_{p}{\left(1+\left(\tau_{fr}^{2}/\tau_{p}^{2}\right)\right)}^{1/2}-\tau_{y}\right)$$

(29) $$\left(\frac{r_{1}Q_{0}^{coh}}{{\hat{\tau }}_{r}^{coh}}+\frac{r_{2}Q_{0}^{ord}}{{\hat{\tau }}_{r}^{ord}}+\frac{r_{3}Q_{0}^{oro}}{{\hat{\tau }}_{r}^{oro}}\right)$$

where

(30) $$Q_{y}=Q_{0}-\frac{\tau_{y}}{r_{1}+r_{2}+r_{3}}\left(\frac{r_{1}Q_{0}^{coh}}{{\hat{\tau }}_{r}^{coh}}+\frac{r_{2}Q_{0}^{ord}}{{\hat{\tau }}_{r}^{ord}}+\frac{r_{3}Q_{0}^{oro}}{{\hat{\tau }}_{r}^{oro}}\right)$$

By deflecting Equation (27), the thermal-kinetic partition can be obtained as:

(31) $$-\frac{\partial Q}{\partial \tau }=V^{*}=\frac{1}{r_{1}+r_{2}+r_{3}}\left(\frac{r_{1}Q_{0}^{coh}}{{\hat{\tau }}_{r}^{coh}}+\frac{r_{2}Q_{0}^{ord}}{{\hat{\tau }}_{r}^{ord}}+\frac{r_{3}Q_{0}^{oro}}{{\hat{\tau }}_{r}^{oro}}\right)$$

with $V^{*}$ as the activation volume.

By rearranging the terms in Equation (29), an expression is given as:

(32) $$\left(\frac{Q-Q_{y}}{Q_{y}}\right){\left(\frac{∆G+\tau_{0}+\tau_{p}{\left(1+\left(\tau_{fr}^{2}/\tau_{p}^{2}\right)\right)}^{1/2}-\tau_{y}}{\tau_{y}}\right)}^{-1}=-\frac{\tau_{y}}{Q_{y}\left(r_{1}+r_{2}+r_{3}\right)}\left(\frac{r_{1}Q_{0}^{coh}}{{\hat{\tau }}_{r}^{coh}}+\frac{r_{2}Q_{0}^{ord}}{{\hat{\tau }}_{r}^{ord}}+\frac{r_{3}Q_{0}^{oro}}{{\hat{\tau }}_{r}^{oro}}\right)$$

For a given PD, the right-hand term in Equation (32) remains constant due to the relevant intrinsic material parameters and the determined properties. As such, Equation (32) fundamentally indicates that, upon uniform PD, an increased ∆G is always accompanied by a decreased Q, which further changes simultaneously in scale. Following a procedure analogous to Refs. [35,44,46,73,74], the GS can be according to Equation (32) derived as:

(33) $$∆=\frac{Q}{Q_{y}}-\frac{∆G+\tau_{0}+\tau_{p}{\left(1+\left(\tau_{fr}^{2}/\tau_{p}^{2}\right)\right)}^{1/2}}{\tau_{y}}$$

with the reference state as ${Q^{*}=Q}_{y}$ and ${∆G}^{*}=\tau_{y}-\left(\tau_{0}+\tau_{p}{\left(1+\left(\tau_{fr0}^{2}/\tau_{p}^{2}\right)\right)}^{1/2}\right)$ at the yielding point, where the GS just equals to 0. The ∆G in Equation (33) will be substantially increased in contrast with the decreased Q, thus decreasing substantially the GS. The GS is feasible to evaluate the kinetic stability considering the thermo-kinetic correlation. It can be deduced from Equations (32) and (33) that, for a fixed reference state, a thermodynamically more stable or more unstable state corresponds to increasing or decreasing the GS (or increasing the absolute value of GS) [35,73,74]. In this work, the thermo-kinetic parameters, ∆G, Q, $V^{*},$ and GS for dislocation are all related to R and $V_{f}$ of CNPs.

## 4. Model Validation

### 4.1. Yield Stress Increment

**Figure 3:**
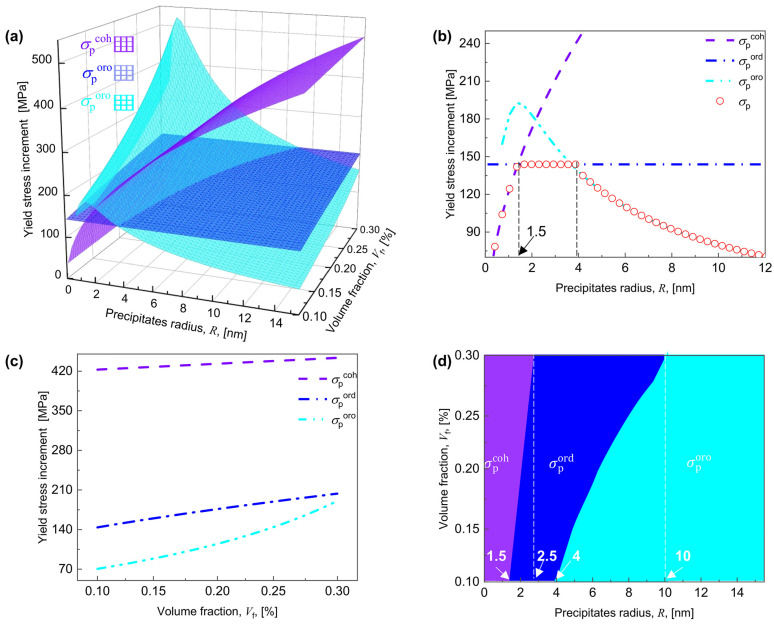
(**a**) Evolution of the theoretical yield stress increment with the radius R and the volume fraction $V_{f}$ of CNPs for coherency strengthening $\sigma_{p}^{coh}$, order strengthening $\sigma_{p}^{ord}$ and Orowan strengthening $\sigma_{p}^{oro}$; (**b**) the longitudinal section parallel to the R axis taken from (**a**) by fixing $V_{f}\;$= 0.1%, in which the symbol of red circle represent the precipitate stress $\sigma_{p}$ determined by $\sigma_{p}=min\left\{\sigma_{p}^{coh},\sigma_{p}^{oro},\sigma_{p}^{ord}\right\}$; (**c**) the longitudinal section parallel to the $V_{f}$ axis taken from (**a**) by fixing R = 12 nm; (**d**) the projection of $\sigma_{p}=min\left\{\sigma_{p}^{coh},\sigma_{p}^{oro},\sigma_{p}^{ord}\right\}$ taken from (**a**) on the $RV_{f}$ axis.

In this work, Equations (S2)–(S4) are utilized to calculate how the shear resistance of precipitates acting on the dislocations evolves with R and $V_{f}$ of the CNPs. Applying the Taylor factor M to convert the shear resistance to the axial yield stress increment by $\sigma_{p}^{x}=M\tau_{p}^{x}$, (x=coh,ord,oro), Figure 3a shows how the yield stress increment varies concerning both R and $V_{f}$. For each given $V_{f},$ the strengthening mechanism follows a sequence of the coherency, the order, and the Orowan-dominated strengthening as R increases. To further investigate the evolution of the strengthening mechanism, the longitudinal section parallel to the R axis was taken from Figure 3a by fixing $V_{f}$ = 0.1%, as shown in Figure 3b. For small R values (R< 1.5 nm), the condition of $\sigma_{p}^{coh}<\sigma_{p}^{ord}<\sigma_{p}^{oro}$ is guaranteed, indicating the coherency-dominated strengthening where the dislocation is impeded by the coherent strain energy. As for 1.5 nm <R< 4.0 nm, the strengthening mechanism is replaced by the order-dominated strengthening, where the dislocation is hindered by the APB, as indicated by the lowest $\sigma_{p}^{ord}$ (see red circle in Figure 3b). Once R exceeds 4 nm, the condition of $\sigma_{p}^{oro}<\sigma_{p}^{ord}<\sigma_{p}^{coh}$ becomes satisfied, and the dislocation bows around the CNPs, demonstrating Orowan-dominated strengthening. The findings coincide with previous studies for Al–0.18Sc [75] and Al–Sc–Zr [76] alloys, where order-dominated strengthening acts as the primary mechanism for 1 nm <R< 3 nm.

In addition, $\sigma_{p}^{coh}$, $\sigma_{p}^{ord},$ and $\sigma_{p}^{oro}$ all grow with increasing $V_{f}$, thus increasing $\sigma_{p}$ for any given R. For instance, in Figure 3c, which is the longitudinal section parallel to the $V_{f}$ axis taken from Figure 3a by fixing R = 12 nm, as $V_{f}$ rises from 0.1% to 0.3%, $\sigma_{p}^{coh}$ increases from 422 to 443 MPa, $\sigma_{p}^{ord}$ grows from 143 to 203 MPa, and $\sigma_{p}^{oro}$ increases from 70 to 190 MPa, resulting in a relationship of ${∆\sigma }_{p}^{oro}$ (120 MPa) > ${∆\sigma }_{p}^{ord}$ (60 MPa) > $∆\sigma_{p}^{coh}$ (21 MPa). Therefore, as $V_{f}$ increases, the critical R values for the transition from coherency to order and from order to Orowan-dominated strengthening increase, e.g., as $V_{f}$ increases from 0.1% to 0.3%, the value of R for the transition from coherency to order-dominated strengthening increases from 1.5 to 2.5 nm, while for the transition from order to Orowan-dominated strengthening increases from 4 to 10 nm (Figure 3d).

### 4.2. Stress-Strain Curve

In this section, MATLAB software (Version: 2018a) is used to program and solve the dislocation-based strain hardening model coupling *R* and $V_{f}$ of the CNPs. To minimize the influence of adjustable parameters in Equation (21), namely, the temperature-related constant C and the atomic vibration frequency $v_{0}$, the dislocation density evolution is assumed as [40,41,77]:

(34) $$\frac{d\rho }{d\gamma }=k_{1c}\sqrt{\rho }-k_{2c}\rho$$

with $k_{1c}$ and $k_{2c}$ as the constant dislocation storage coefficient and the constant kinetic recovery coefficient. Then, the flow stress can be according to Ref. [77] obtained as:

(35) $$\sigma =\sigma_{i}+\left(\sigma_{\infty }-\sigma_{i}\right)\left(1-exp\left(-\frac{\varepsilon }{\tilde{\varepsilon }}\right)\right)$$

where

(36) $$\sigma_{\infty }=\sigma_{p}+\frac{M\alpha G_{m}bk_{1c}}{k_{2c}}$$

(37) $$\tilde{\varepsilon }=\frac{2}{Mk_{2c}}$$

(38) $$\sigma_{i}=\sigma_{0}+{\left(\sigma_{p}^{2}+{\left(\alpha {MG}_{m}b\sqrt{\rho_{0}}\right)}^{2}\right)}^{1/2}$$

**Figure 4:**
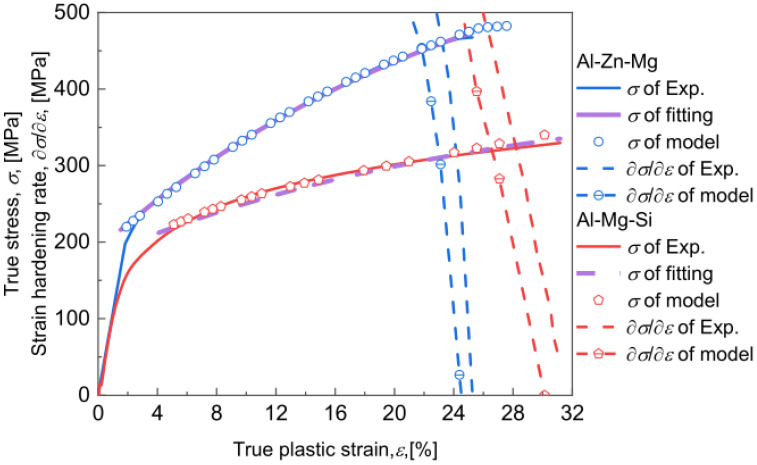
True stress-strain curves and strain hardening rate of experiment data and model prediction for Al-Zn-Mg alloy with R = 0.8 nm and $V_{f}$ = 1.1% of nanoclusters [78] and for Al-Mg-Si alloy with R = 4 nm and $V_{f}$ = 0.1% of CNPs, and the fitting curves of Equation (35).

Fits of Equation (35) to the experimental data are performed, and the results (purple solid and dashed lines) are shown in Figure 4. The main fitting parameters are listed in Table 3. The experimental stress-strain curve due to Al-Zn-Mg alloy naturally aged for 1440 h with R = 0.8 nm and $V_{f}$ = 1.1% of nano-clusters [78] is labeled by the blue solid line, while that due to Al-Mg-Si alloy naturally aged for 1440 h with R = 4 nm and $V_{f}$ = 0.1% of CNPs is labeled by the red solid line.

Values for R and $V_{f}$ of CNPs are counted from the TEM image shown in Figure S1. Following the calculation scheme summarized in Figure S2 in the Supplementary Materials, first, the stress-strain curve by tensile test can be used to determine the constant values of $k_{1c}$ and $k_{2c}$ (Table 3) by fits of the stress-strain response equation (Equation (29)) integrated by the classical K-M equation. Values for $V_{f}$ and R, as well as the initial dislocation density $\rho_{0}$ of undeformed samples, can be determined using TEM and HEXRD, so that $\tau_{p}$, $\tau_{f0},$ and $\tau_{r}$ can be calculated by Equations (S2)–(S4) in the Supplementary Materials and Equation (14). Thereafter, two adjustable parameters C and $v_{0}$ can be calculated using Equations (24) and (25). For the Al-Zn-Mg and the Al-Mg-Si alloy, respectively, C = −51.17 and $v_{0}$ = 2.3 × 10^11^ s^−1^, and C = −72.5 and $v_{0}$ = 2.32 × 10^11^ s^−1^, were obtained; the values for C fall within the range of about −25 to −200 at temperatures ranging from 0 and 1000 K [42], while the values for $v_{0}$ align with the order of ~10^11^ s^−1^ of the instantaneous unpinning rate at an obstacle [51]. For a specific combination of $V_{f}$ and R, $\tau_{p}$, $\tau_{f},$ and $\tau_{r}$ for various ε corresponding to various ρ are determined by Equations (S2)–(S4) and (14). Equations (21)–(33) are used to calculate the stress-strain curve, the ρ, and the thermo-kinetic parameters, through the ode23 function package of MATLAB software.

Values for model parameters used to calculate the stress-strain curve are shown in Table 4. The calculated stress-strain curves of Al-Zn-Mg alloy and Al-Mg-Si alloy are presented as blue circles and red pentagons in Figure 4, where the strain hardening rate ∂σ/∂ε is derived using numerical differentiation performed for stress-strain curves, and as one can see, the present model accurately predicts the macroscopic strain hardening experimentally deduced. Furthermore, a good agreement between the experimentally measured necking strain $\varepsilon_{n}$ (24.2% for Al-Zn-Mg alloy and 26.1% for Al-Mg-Si alloy) and the model-predicted $\varepsilon_{n}$ (23.1% for Al-Zn-Mg alloy and 25.7% for Al-Mg-Si alloy) results evidently. It is worth noting that in this paper, in addition to fitting parameters (Table 3) and general parameters (Table 4), the kinetic parameters, i.e., the $Q^{coh}$, $Q^{ord},$ and $Q^{oro}$, the ${\hat{\tau }}_{r}^{ord}$, ${\hat{\tau }}_{r}^{coh},$ and ${\hat{\tau }}_{r}^{oro}$ and the $r_{1}$, $r_{2},$ and $r_{3}$, are all calculated by $V_{f}$ and R of CNPs.

### 4.3. Dislocation Density

**Figure 5:**
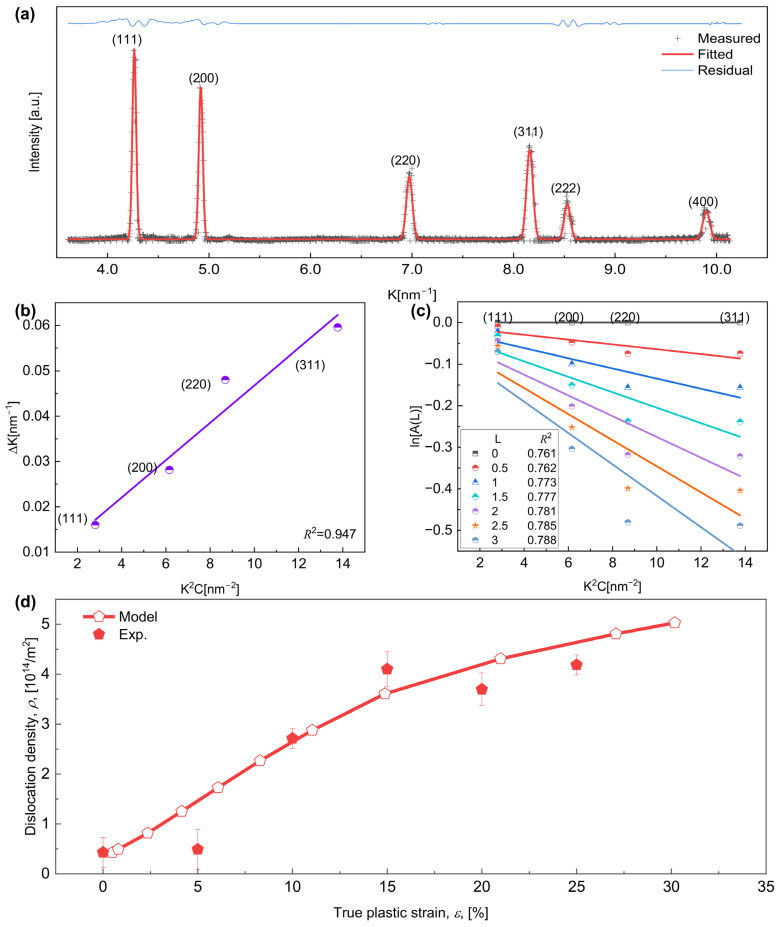
Calculation of dislocation density. (**a**) The measured (black “+” marks) and fitted (solid red line) high-energy X-ray diffraction (HEXRD) profiles of undeformed specimen with R = 4 nm and $V_{f}$ = 0.1% of CNPs. The residual between measured and fitted profiles is plotted in a solid blue line. K is the reciprocal of the lattice spacing. (**b**) Modified Williamson–Hall plot and (**c**) Modified Warren–Averbach plot obtained from the peaks of α-Al phase in the fitted HEXRD profile shown in (**a**). (**d**) Evolution of the dislocation density ρ of α-Al with the true plastic strain ε calculated from HEXRD profiles and from the present model.

The HEXRD profiles of samples with different true plastic strains are shown in Figure S5. The modified Williamson–Hall (MWH) method and the modified Warren–Averbach (MWA) method were combined to calculate the average dislocation densities of all specimens based on the 1-D HEXRD profiles [48,79]. Using the undeformed specimen as an example, the measured 1-D HEXRD profiles were first fitted using the pseudo-Voigt function [80], as shown in Figure 5a. The residual, shown by a solid blue line between the measured (black “+” marks) and fitted (solid red line) profiles, is negligibly small, which indicates a good fitting. Four α-Al phase peaks, including (111), (200), (220), and (311) shown in Figure 5a, were used for the calculation of average dislocation densities of the α-Al phase. The MWH and MWA plots calculated based on the α-Al phase peaks are shown in Figure 5b and Figure 5c, respectively. It can be seen that the goodness-of-fit values, i.e., *R*^2^, of the fitting curves are all greater than 0.7, which indicates that the fitting results are accurate. Before PD, the initial dislocation density of α-Al phase is calculated to be 4.27 × 10^13^ m^−2^. This value is in excellent agreement with α-Al phase dislocation density of about 1 × 10^13^ m^−2^ that was calculated by convolutional multiple global contours (CMWP) method, previously reported in the natural aging of Al-Zn-Mg alloy [78,81].

Evolution of the dislocation density assuming R = 4 nm and $V_{f}$ = 0.1% of CNPs for present samples deformed at different strains were performed, where the tensile tests were quit at true plastic strain levels of 5%, 10%, 15%, 20%, and 25%, respectively. The calculated results for different true plastic strain ε are displayed in Figure 5d, in which the α-Al phase dislocation density increases from 4.27 × 10^13^ m^−2^ to 5 × 10^14^ m^−2^ as ε increases. This is consistent with the evolution trend of dislocation density measured by synchrotron radiation [78], further proving the accuracy of the model.

## 5. Model Prediction

### 5.1. Prediction of Stress-Strain Responses

**Figure 6:**
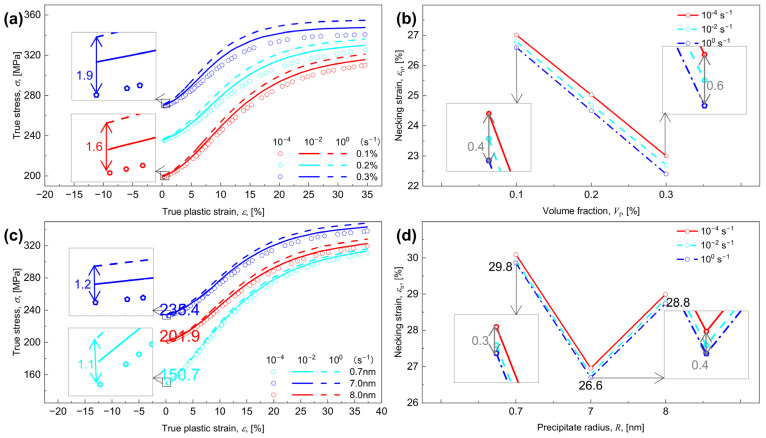
Evolution of the stress-strain responses with the true plastic strain ε for various combinations of volume fraction $V_{f}$, radius R, and strain rate $\dot{\gamma }$. (**a**) The true stress σ vs. ε for various combinations of $V_{f}$ and $\dot{\gamma }$; (**b**) the necking strain $\varepsilon_{n}$ vs. $V_{f}$ for various $\dot{\gamma }$; (**c**) the σ vs. ε for various combinations of R and $\dot{\gamma }$, and (**d**) the $\varepsilon_{n}$ vs. R for various $\dot{\gamma }$. The inserts in (**a**) represent the enlarged drawings at $V_{f}$ = 0.1% (red) and at $V_{f}$ = 0.3% (blue), the inserts in (**b**) represent the enlarged drawings at $V_{f}$ = 0.1% (left) and at $V_{f}$ = 0.3% (right), the inserts in (**c**) represent the enlarged drawings at R = 0.7 nm (cyan) and at R = 7 nm (blue), and the inserts in (**d**) represent the enlarged drawings at R = 0.7 nm (left) and at R = 7 nm (right) for various $\dot{\gamma }$.

Stress-strain curves and necking strain with varying $V_{f}$ for R = 3 nm and varying R for $V_{f}$ = 0.2% performed at three strain rates of 10^−4^, 10^−2^, and 10^0^ s^−1^ are calculated and shown in Figure 6, where the value of $\varepsilon_{n}$ is determined by Hart’s criterion [60]. As generally expected, increasing $V_{f}$ or $\dot{\gamma }$ enhances $\sigma_{y}$ (*ε* = 0) but reduces $\varepsilon_{n}$ (Figure 6a,b). As R increases, $\sigma_{y}$ (*ε* = 0) first grows and then drops, whereas $\varepsilon_{n}$ first declines and then increases, in contrast to the monotonically increased $\sigma_{y}$ with increasing $V_{f}$ at a given $\dot{\gamma }$. As shown clearly, for $\dot{\gamma }$ = 10^0^ s^−1^, the value of $\sigma_{y}$ first grows from 150.7 MPa to 235.4 MPa and then to 201.9 MPa (Figure 6c) and the value of $\varepsilon_{n}$ reduces from 29.8% to 26.6% and then to 28.8% (Figure 6d), as R increases from 0.7 nm to 7 nm and then to 8 nm. Following Equation (26), as $V_{f}$ or R changes, the value of $\sigma_{y}$ is dominated by $\sigma_{p}$, since the initial dislocation density $\rho_{0}$ and the lattice resistance $\tau_{0}$ remain constant independent of $V_{f}$ and R. Increasing $V_{f}$ results in higher values of $\sigma_{p}^{coh}$, $\sigma_{p}^{ord},$ and $\sigma_{p}^{oro}$, which in turn leads to an increase in $\sigma_{p}$ (Figure 3c) and subsequently a rise in $\sigma_{y}$. As R increases, the precipitate strengthening mechanism shifts from coherency to order and finally Orowan-dominated strengthening, so that $\sigma_{p}$ (Figure 3b) and $\sigma_{y}\;$(Figure 6c) first rises and subsequently falls.

Upon observing the inserts in Figure 6a,b, clearly, as $V_{f}$ increases from 0.1% to 0.3%, the increment of $\sigma_{y}$ with varying $\dot{\gamma }$ from 10^−4^ to 10^0^ s^−1^ increases from 1.6 MPa to 1.9 MPa. Additionally, the reduction of $\varepsilon_{n}$ increases from 0.4% to 0.6%. See the inserts in Figure 6c,d, as R increases from 0.7 nm to 7 nm, the increment of $\sigma_{y}$ for $\dot{\gamma }$ ranging from 10^−4^ to 10^0^ s^−1^ goes from 1.1 MPa to 1.2 MPa and the reduction of $\varepsilon_{n}$ increases from 0.3% to 0.4%. Both instances illustrate that the change of $\sigma_{y}$ and $\varepsilon_{n}$ for varied $\dot{\gamma }$ become more noticeable, as $V_{f}$ or R increases.

### 5.2. Prediction of Strain Hardening and Strain-Rate Hardening

**Figure 7:**
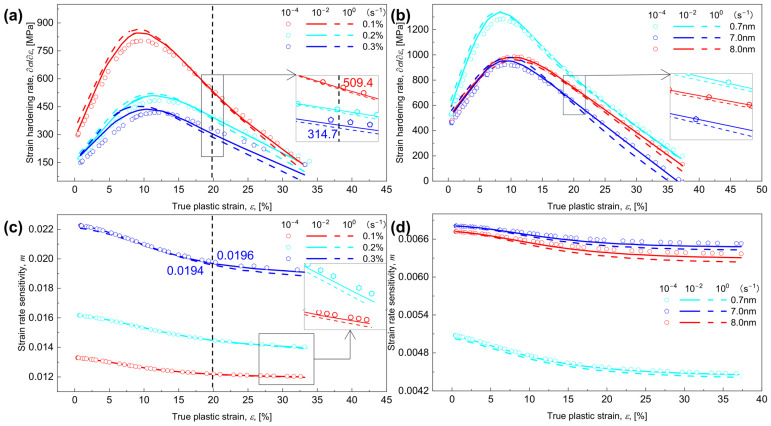
Evolution of the strain hardening rate ∂σ/∂ε  and the strain rate sensitivity m with the true plastic strain ε for various combinations of volume fraction $V_{f}$, radius R, and strain rate $\dot{\gamma }$. The ∂σ/∂ε vs. ε for (**a**) various $V_{f}$ and (**b**) various R; and the m vs. ε for (c) various $V_{f}$ and (d) various R performed at three $\dot{\gamma }$ of 10^−4^, 10^−2^, and 10^0^ s^−1^.

According to the necking instability criterion [41,60], the evolution of $\varepsilon_{n}$ depends on the strain hardening rate and the strain rate sensitivity m [48,78]. As expected, an increased $V_{f}$ or $\dot{\gamma }$ accelerates the occurrence of necking point by descreasing the strain hardening rate, which can be seen in Figure 7a, where, as $V_{f}$ rises from 0.1% to 0.3%, the strain hardening rate at ε = 20% falls from 509.4 to 314.7 MPa for $\dot{\gamma }$ = 10^−4^ s^−1^. A similar trend can be seen in Ref. [31], where, for shearing-dominated strengthening, the increased $V_{f}$ from 0.4% to 0.6% makes the strain hardening rate decrease from 3000 MPa to 1000 MPa. As described in Section 4.1, the increase of R will change the interaction between dislocations and CNPs from shearing to Orowan-dominated strengthening. As R increases from 0.7 to 7 nm (see the insert in Figure 7b), the predominant shearing mechanism reduces the strain hardening rate, thus leading to a drop in $\varepsilon_{n}$, whereas, as R increases from 7 to 8 nm, the predominant Orowan mechanism enhances the strain hardening rate, thus leading to an increase in $\varepsilon_{n}$.

Furthermore, the strain rate sensitivity, m is calculated and shown in Figure 7c,d, where, the increased $\dot{\gamma }$ decreases the m, e.g., for $V_{f}$ = 0.3%, the value of m at a strain of 20% decreases from 0.0196 to 0.0194 with $\dot{\gamma }$ increases from 10^−4^ to 10^0^ s^−1^, while for any given $\dot{\gamma }$, m reduces with increasing ε, see also Ref. [82]. This can also be inferred from Figure 6a,c that, independent of $V_{f}$ and R, the decreased value of m tends to enhance $\sigma_{y}$, as $\dot{\gamma }$ increases; see also [48,83]. Analogously, as $\dot{\gamma }$ increases, the enhanced stress level (Figure 6a) and the declined strain level (Figure 6b) due to the increased m (Figure 7c) will become expanded, and moreover, a continuously increased $V_{f}$ makes m increase steadily (Figure 7c), but with increasing R, m first grows and subsequently declines (Figure 7d).

### 5.3. Predictions of Dislocation-Related Variables

**Figure 8:**
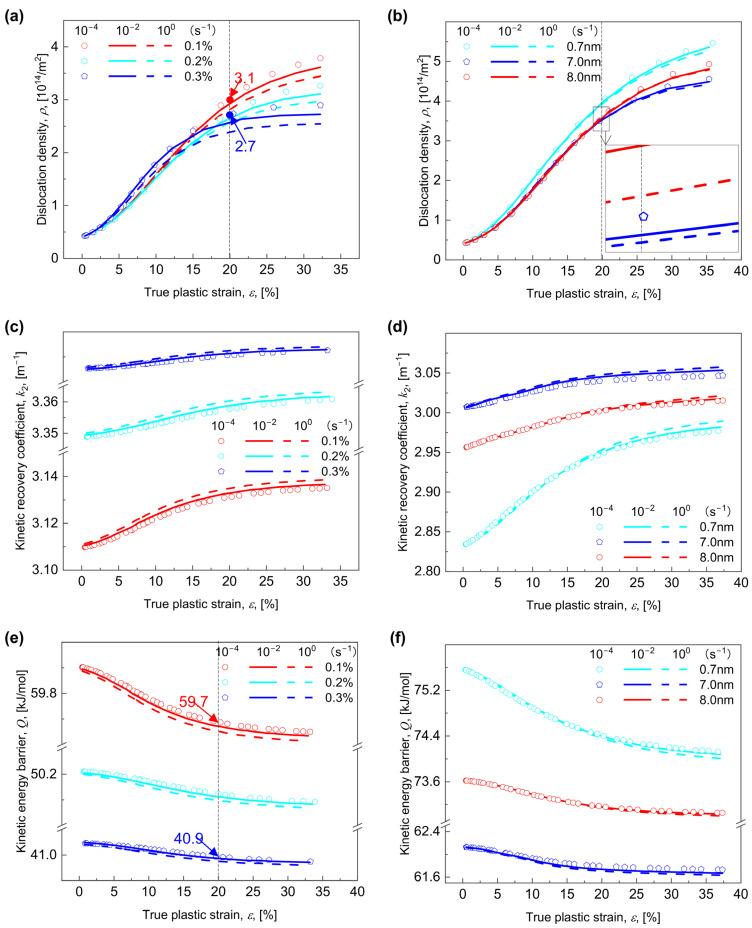
Evolution of the dislocation-related variables with the true plastic strain ε for various combinations of volume fraction $V_{f}$, radius R, and strain rate $\dot{\gamma }$. The dislocation density ρ vs. ε for (**a**) various $V_{f}$ and (**b**) various R; the kinetic recovery coefficient $k_{2}$ vs. ε for (**c**) various $V_{f}$ and (**d**) various R; and the kinetic energy barrier Q vs. ε for (**e**) various $V_{f}$ and (**f**) various R performed at three $\dot{\gamma }$ of 10^−4^, 10^−2^, and 10^0^ s^−1^.

Physically, the strain hardening rate is decided by the forest dislocation density ρ [5,40]. The dislocation-related variables with various $V_{f}$ for R = 3 nm and various R for $V_{f}$ = 0.2% performed at three $\dot{\gamma }$ of 10^−4^, 10^−2^, and 10^0^ s^−1^ are calculated and shown in Figure 8, where, for any combination of $V_{f}$, R, and $\dot{\gamma }$, generally, ρ grows as ε increases; see similar tendency observed for the Al-Zn-Mg alloy [78]. As expected, ρ reduces with rising $V_{f}$ (Figure 8a), where, for a $\dot{\gamma }$ of 10^−4^ s^−1^,the value of ρ at 20% plastic strain falls from 3.1 × 10^14^ m^−2^ to 2.7 × 10^14^ m^−2^ as $V_{f}$ rises from 0.1% to 0.3%; whereas, analogous to Figure 6 and Figure 7, ρ exhibits a typical pattern of decreasing first and then increasing as R grows (see Figure 8b).

Analogous to the evolution of stress, strain, and strain rate sensitivity, the continuously increased $V_{f}$ always makes $k_{2}$ increase steadily (Figure 8c), but with increasing R, $k_{2}$ first grows and subsequently declines (Figure 8d). With reference to Equation (21), actually, the decline in ρ determines the rise in $k_{2}$, which is mainly correlated with the Q for dislocation gliding. Equation (25) states that $k_{2}$ is inversely proportional to Q, namely, it decreases with increasing Q. As for $\dot{\gamma }$ = 10^−4^ s^−1^, the value of Q for ε = 20% decreases from 59.7 to 40.9 kJ/mol as $V_{f}$ increases from 0.1% to 0.3% (Figure 8e). If both $\dot{\gamma }$ and $V_{f}$ remains constant, Q will first fall and then increase as R increases (Figure 8f). This is analogous to the level of kinetic energy barrier of dislocation gliding determined by the nanoindentation [52]. The above findings indicate that the rise in $\varepsilon_{n}$ is attributable to an increase in ρ, which is related to a decrease in $k_{2}$, which is in turn connected to an increase in Q, independent of $V_{f}$, $\dot{\gamma }$, or R.

## 6. Discussion

As described in Section 5, several so-called trade-off phenomena can be summarized for a single or multiple PDs, so their physical origin deserves detailed studies. On this basis, it will be more interesting to find how to break these trade-off limits, depending on the present cooperative strengthening of multiple interactions. If so, R and $V_{f}$ will be designed by optimizing the trade-off between $\sigma_{y}$ and $\varepsilon_{n}$.

### 6.1. Trade-Off Relationships

#### 6.1.1. Trade-Off Relationships in Single PD

**Figure 9:**
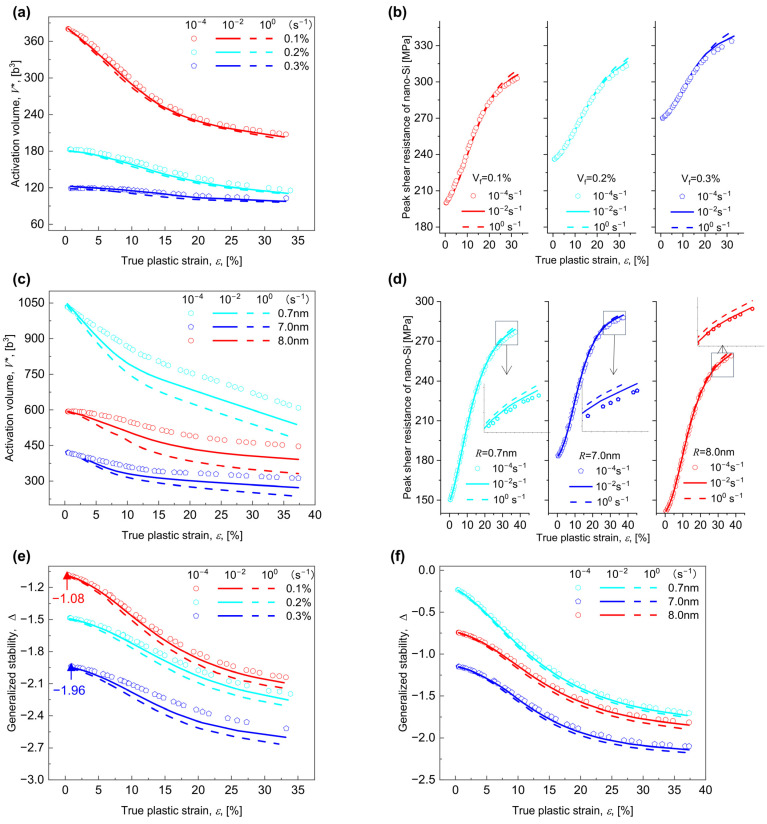
Evolution of (**a**) the activation volume $V^{*}$ and (**b**) the peak shear resistance for various combinations of volume fraction $V_{f}$ and strain rate $\dot{\gamma }$, (**c**) the $V^{*}$ and (**d**) the peak shear resistance for various combinations of radius R and $\dot{\gamma }$, and the generalized stability for various combinations of (**e**) $V_{f}$ and $\dot{\gamma }$ and (**f**) R and $\dot{\gamma }$ with the true plastic strain ε.

As a basic knowledge, the trade-off relationship between the strain hardening rate and the strain prevails in a single PD, i.e., the strain hardening rate generally declines as ε increases until it falls below the applied stress. It is stated in Section 5.3 that the reduced strain hardening rate can be attributed to the increased $k_{2}$, as a consequence of the decreased Q. In a single PD, the trade-off between strain hardening rate and ε is attributed to the trade-off between Q and ε. Applying the current model, the trade-off between Q and ε can be quantitatively analyzed by calculating Equation (31); see the decreasing $V^{*}$ with increasing ε in Figure 9a, while keeping invariable $V_{f}$, R, and $\dot{\gamma }$. This fits well with the experiment findings of Ref. [82], where $V^{*}$ decreases with increasing ε for any R and $\dot{\gamma }$.

Combined with Figure 7c and Figure 8e, for any given PD, m, $V^{*},$ and Q all drop as ε increases, which seems to contradict the traditional trade-off relationship between m and $V^{*}$ [51,82,84]. According to $m=k_{B}T/V^{*}\tau$, m is actually connected to both $V^{*}$ and τ in a single PD, and further following $Q=Q_{0}-V^{*}\tau$ yields $m=k_{B}T/\left(Q_{0}-Q\right)$. As determined by $Q_{0}=\left(r_{1}Q_{0}^{ord}+r_{2}Q_{0}^{coh}+r_{3}Q_{0}^{oro}\right)/\left(r_{1}+r_{2}+r_{3}\right)$ in Equation (28), m and Q are positively connected, so that m, $V^{*},$ and Q all decrease as ε increases in a single PD. Noted that $r_{1}$, $r_{2},$ and $r_{3}$ in Equation (31) strands for the contribution due to three strengthening mechanisms, which are shown in Figures S3 and S4 in the Supplementary Materials. As for a combination of R = 3 nm, $V_{f}$ = 0.3%, $\dot{\gamma }$ = 10^0^ s^−1^, according to Figure 3d, the order strengthening dominanted. Once the dislocation glide begins, for example, a relation of $r_{2}>r_{1}>r_{3}$ always holds (Figure S3i), indicating that the order strengthening plays a dominant role in the dislocation gliding kinetics. As ε increases, nevertheless, $r_{2}$ and $Q_{0}^{ord}$ remain almost constant, so that the decrease of $V^{*}$ arises mainly from the increase in peak resistance induced by the order strengthening ${\hat{\tau }}_{r}^{ord}$ (Figure 9b), thus declining the strain hardening rate.

#### 6.1.2. Trade-Off Relationships in Multiple PDs

For multiple PDs assuming different combinations of $V_{f}$, R, and $\dot{\gamma }$, the increased $\sigma_{y}$ is always accompanied by the decreased $\varepsilon_{n}$, reflecting the trade-off relationship between $\sigma_{y}$ and $\varepsilon_{n}$. This basically depends on the evolution of $V^{*}$. As illustrated in Figure 9b, for $\dot{\gamma }$ = 10^−4^ s^−1^, under all the three $V_{f}$ conditions, the maximum value of peak resistance increases from 315 to 337 MPa, and in combination with ${\hat{\tau }}_{r}^{ord}\propto \tau_{p}^{ord}\propto \sqrt{V_{f}}$ (Equation (S3)), the decrease in $V^{*}$ with the increase in $V_{f}$ is attributed to the increase in ${\hat{\tau }}_{r}^{ord}$. As shown in Figure 9c, $V^{*}$ first declines and subsequently grows as R increases, which can be attributed to the transition from the shearing to the Orowan-dominated mechanism; see Section 5.1. From Figure S4a–c, it can be seen that, for the shearing-dominated mechanism, i.e., R=0.7 nm, the prevalence of $r_{1}>r_{2}>r_{3}$ implies that dislocations and CNPs interact by the coherency strengthening, and ${\hat{\tau }}_{r}^{coh}\propto \tau_{p}^{coh}\propto \sqrt{R}$ (Equation (S2)) holds, i.e., as R increases, ${\hat{\tau }}_{r}^{coh}$ increases (Figure 9d), thus decreasing $V^{*}$. From Figure S4d–i, it can be seen that, for the Orowan-dominated mechanism, i.e., R=7 and 8 nm, the prevalence of $r_{3}>r_{2}>r_{1}$ implies that dislocation and CNPs interact by the Orowan mechanism, and ${\hat{\tau }}_{r}^{oro}\propto \tau_{p}^{oro}\propto lnR/R$ (Equation (S4)) holds, i.e., as R increases, ${\hat{\tau }}_{r}^{oro}$ decreases (Figure 9d), thus increasing $V^{*}$. This is further evidenced in Figure 7d and Figure 8f, where the trade-off relationship between m and $V^{*}$ is satisfied [51], independent of *R*.

After breaking the TS, a reduced ${\hat{\tau }}_{r}$ leads to an increased $V^{*}$, which increases the Q, and thus reduces the velocity and, in turn, improves the sustainability for dislocation gliding, i.e., enhancing the corresponding GS [35,44,46,71]. The GS for dislocation gliding calculated by Equation (33) is shown in Figure 9e,f. As for the $\dot{\gamma }$ of 10^−4^ s^−1^, the GS gradually decreases with increasing $V_{f}$, e.g., from −1.08 to −1.96 with $V_{f}$ from 0.1% to 0.3% (Figure 9e). Physically, the increased $V_{f}$ favors the increased ${\hat{\tau }}_{r}$ (Figure 9b), and then supports the reduced $V^{*}$ (Figure 9a) and the decreased Q (Figure 8e), thus descending the GS for dislocation gliding (Figure 9e), corresponding to an increased velocity and $k_{2}$ (Figure 8c). This inevitably gives an unsustainable strain hardening and consequently reduces the $\varepsilon_{n}$ (Figure 6b). However, as R increases for a certain $\dot{\gamma }$, the interactions between CNPs and dislocations are transferred from the shearing to the Orowan mechanism, which leads to an initially increased but subsequently decreased ${\hat{\tau }}_{r}$ (Figure 9d), and in turn, the initially decreased but lately increased $V^{*}$ (Figure 9c) and Q (Figure 8f). In this way, the GS (Figure 9f) for dislocation gliding declines first and then grows, which first increases and then decreases the velocity and $k_{2}$ (Figure 8d), corresponding to an initially dropped but subsequently enhanced $\varepsilon_{n}$ (Figure 6d).

### 6.2. How to Break Trade-Offs

#### 6.2.1. Cooperative and Individual Strengthening

**Figure 10:**
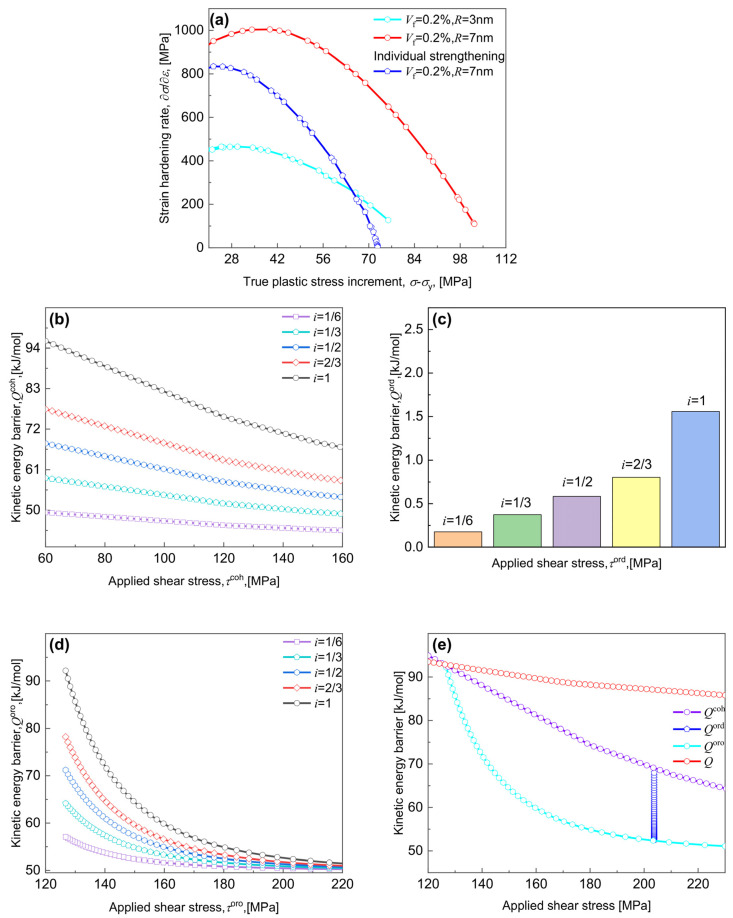
(**a**) Evolution of the strain hardening rate ∂σ/∂ε with the true plastic stress increment ($\sigma -\sigma_{y}$) for cases by combinations of a cooperative strengthening of coherency, order, and Orowan mechanism with R = 3 and 7 nm, and of an individual strengthening of the Orowan mechanism with R = 7 nm. Evolution of the kinetic energy barrier with the applied shear stress for (**b**) coherency mechanism, (**c**) order mechanism, (**d**) Orowan mechanism under various i values, and (**e**) three mechanisms with cooperative strengthening and individual strengthening under i = 1.

For CNPs-strengthened alloys, the cooperative strengthening due to multiple interactions between dislocations and CNPs is usually observed upon PDs [11,12,85]. As mentioned in Section 1, typical models focusing on the individual strengthening of Orowan or shearing mechanism seem not appropriate for alloys strengthened by the cooperative mechanisms [33,34]. This can be clearly demonstrated in Figure 10a, where the model-calculated strain hardening rates were shown, including R = 3 and 7 nm with $V_{f}$ = 0.2% controlled by the cooperative strengthening of coherency, order, and Orowan mechanism, and R = 7 nm with $V_{f}$ = 0.2% controlled by the individual strengthening of the Orowan mechanism. The model that considers the individual strengthening can be considered as a simplification of Equation (31), so that, once the value of $\sigma -\sigma_{y}$ exceeds 65 MPa, the strain hardening rate for the individual strengthening becomes lower than that for shearing-dominated strengthening, consistent with previous findings [30,31,32].

To predict the Q vs. τ relationship in the regime where thermal activation plays a major role ($\tau /{\hat{\tau }}_{r}$< 1), the Q considering the individual strengthening and the cooperative strengthening has been herein derived (Equation (S7) and Equation (23)). Figure 10b–d shows the predictions performed for $Q^{coh}$ vs. $\tau^{coh}$, $Q^{ord}$ vs. $\tau^{ord},$ and $Q^{oro}$ vs. $\tau^{oro}$ by altering the R while assuming $V_{f}$ = 0.1%. As described below in Equation (S7) in the Supplementary Materials, the physical parameter i in the present model appears to be mathematically equivalent to the adjustable parameter p with q = 1 in the K-M model. Reducing the value of i tends to reduce the values for $Q^{coh}$, $Q^{ord},$ and $Q^{oro}$ at the same level for applied shear stress (Figure 10b–d). This suggests that randomizing obstacles away from typical evenly spaced distribution may reduce the kinetic energy barrier for dislocation gliding. Furthermore, Figure 10e predicts the relationships for $Q^{coh}$ vs. $\tau^{coh}$, $Q^{ord}$ vs. $\tau^{ord}$, $Q^{oro}$ vs. $\tau^{oro},$ and Q vs. τ with i = 1. As the applied stress increases, both $Q^{coh}$ and $Q^{oro}$ fall, particularly the $Q^{oro}$ showing a substantial reduction; for the prevalence of the order strengthening, typically, the $\tau^{ord}$ reaches a constant (Figure 3b), as associated with the constant value of APB during dislocation shearing CNPs, but in contrast with the increased $Q^{ord}$ with the increased R. Therefore, it can be implied from Figure 10e that a higher effective Q under the same stress level could be obtained, once the cooperative strengthening is considered, in substitution of the individual strengthening as $Q^{coh}$, $Q^{ord},$ or $Q^{oro}$.

#### 6.2.2. Breaking Trade-Offs by Enhancing Activation Volume

**Figure 11:**
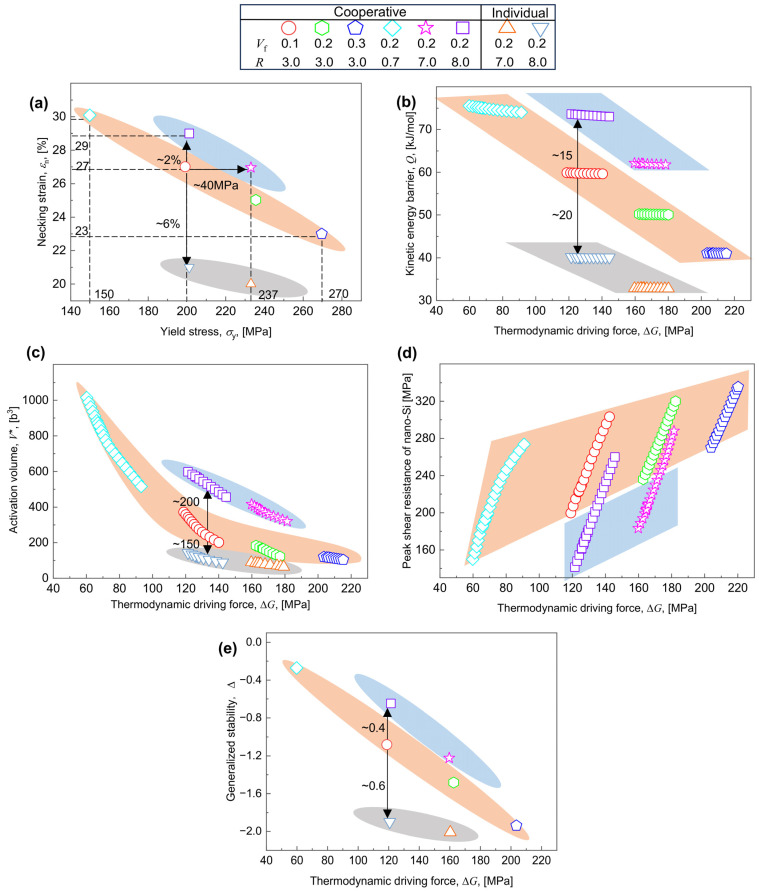
Evolution of (**a**) the necking strain $\varepsilon_{n}$ with the yield stress $\sigma_{y}$, (**b**) the kinetic energy barrier Q, (**c**) the activation volume $V^{*}$, (**d**) the peak resistance, and (**e**) the generalized stability with the thermodynamic driving force ∆G for various combinations of volume fraction $V_{f}$ and radius R due to cooperative strengthening of shearing (orange ellipse) and of the Orowan-dominated mechanism (blue ellipse), and due to individual strengthening of the Orowan-dominated mechanism (gray ellipse).

Figure 11a displays the estimated values of $\sigma_{y}$ and $\varepsilon_{n}$ assuming various combinations of $V_{f}$ and R for cooperative strengthening and individual strengthening of the Orowan mechanism. A negative correlation between $\sigma_{y}$ and $\varepsilon_{n}$ forms evidently in the orange ellipse, e.g., as $\sigma_{y}$ increases from 150 to 270 MPa, $\varepsilon_{n}$ decreases from 30% to 23%. In this situation, the interaction between CNPs and dislocations favors the shearing-dominated mechanism (including the coherency and the order-dominated mechanism). As for the Orowan-dominated mechanism (the blue ellipse), the trade-off between $\sigma_{y}$ and $\varepsilon_{n}$ is also illustrated by the decreased $\varepsilon_{n}$ from 29% to 27% with increasing $\sigma_{y}$ from 200 to 237 MPa. Noted that the trade-off level between $\sigma_{y}$ and $\varepsilon_{n}$ due to the Orowan-dominated mechanism is higher than that due to the shearing-dominated mechanism. This contradicts previous research [30,31,32], which reported a reduced trade-off between $\sigma_{y}$ and $\varepsilon_{n}$ due to the individual Orowan strengthening; see also the gray ellipse in Figure 11a. When comparing the mechanical properties assuming $V_{f}$ = 0.1% and R = 3 nm to that assuming $V_{f}$ = 0.2% and R = 8 nm for the cooperative strengthening, a ~2% increase in $\varepsilon_{n}$ without a corresponding decrease in $\sigma_{y}$ can be observed, whereas as compared to those assuming $V_{f}$ = 0.2% and R = 7 nm for the cooperative strengthening, a ~40 MPa increase in $\sigma_{y}$ without a corresponding decrease in $\varepsilon_{n}$ can be seen; see Figure 11a. Such concurrent increases for $V_{f}$ and R (as indicated by the transition between the orange ellipse and the blue ellipse) tend to increase $\sigma_{y}$ and $\varepsilon_{n}$ simultaneously, which implies that the trade-off relationship between $\sigma_{y}$ and $\varepsilon_{n}$ can be broken, only by being subjected to the cooperative strengthening.

As such, the break in trade-off limitation between $\sigma_{y}$ and $\varepsilon_{n}$ reflects the break in thermo-kinetic correlation [35,44,46,71]. Equation (26) illustrates that the deviation of ∆G from (static) equilibrium is affected by the $\tau_{r}$ determining the $\sigma_{y}$ [34]. The model-calculated ∆G and Q are displayed in Figure 11b, where the trade-off relationships between ∆G and Q remain when subjected to PDs, assuming varying combinations of $V_{f}$ and R, are consistent with the trade-off between $\sigma_{y}$ and $\varepsilon_{n}$. Correspondingly, the trade-off between ∆G and Q for the Orowan-dominated mechanism is higher than that for the shearing-dominated mechanism. As described in Section 6.1, the evolution of Q can be attributed to the $V^{*}$. Figure 11c shows a higher trade-off relationship between $V^{*}$ and ∆G due to the Orowan-dominated mechanism (blue area) as compared to the shearing-dominated mechanism (orange area); as for individual mechanism, Equation (31) simplifies to $V^{*}=Q_{0}^{oro}/{\hat{\tau }}_{r}^{oro}$, the corresponding trade-off line between $V^{*}$ and ∆G significantly reduce (the gray ellipse in Figure 11c), decreasing the trade-off line between Q and ∆G (the gray ellipse in Figure 11b), and reducing the strain hardening rate (Figure 10a). The results are in agreement with those published elsewhere [30,31,32]. On this basis, one can see that the breaking of the thermo-kinetic correlation can be attributed to the changed thermo-kinetic partition or $V^{*}$ [82,84]. Further considering the contribution of ${\hat{\tau }}_{r}$ to $V^{*}$ (Section 6.1), Figure 11d shows the ${\hat{\tau }}_{r}$ assuming different combinations of R and $V_{f}$, where, the ${\hat{\tau }}_{r}$ increases with ∆G, whether it is the shearing (orange) or the Orowan-dominated mechanism (blue), since the ${\hat{\tau }}_{r}$ for the Orowan strengthening is always lower than that for the shearing strengthening for comparable ∆G, thus improving the trade-off of the GS and ∆G (Figure 11e).

Comparing the thermo-kinetics assuming $V_{f}\;$= 0.1% and R = 3 nm to that assuming $V_{f}$ = 0.2% and R = 8 nm for the cooperative strengthening, a ~200 b^3^ increase in $V^{*}$ gives a ~15 kJ/mol rise in Q and a ~0.4 improvement in GS without a corresponding decrease in ∆G, providing a ~2% enhancement in $\varepsilon_{n}$ without a corresponding decrease in $\sigma_{y}$; whereas, as compared to that assuming $V_{f}$ = 0.2% and R = 8 nm for the individual strengthening, a ~150 b^3^ decrease in $V^{*}$ causes a ~20 kJ/mol decline in Q and a ~0.6 reduction in GS without a corresponding increase in ∆G, providing a ~6% drop in $\varepsilon_{n}$ without a corresponding increase in $\sigma_{y}$. This further suggests that the trade-off relationship between $\sigma_{y}$ and $\varepsilon_{n}$ can be broken only by increasing $V^{*}$ for cooperative strengthening.

### 6.3. Designing R and $V_{f}$ by Optimizing Trade-Offs

This work intends to develop a dislocation-based strain hardening model that explicitly includes the characteristics of CNPs, so that it can be combined with Hart criterion to describe the trade-off relationship between $\sigma_{y}$ and $\varepsilon_{n}$, and then to optimize the combination of $V_{f}$ and R to simultaneously increase $\sigma_{y}$ and $\varepsilon_{n}$. Taking CNPs of the Al-Mg-Si alloys as an example, a brief synopsis has been shown in Section 4.2.

**Figure 12:**
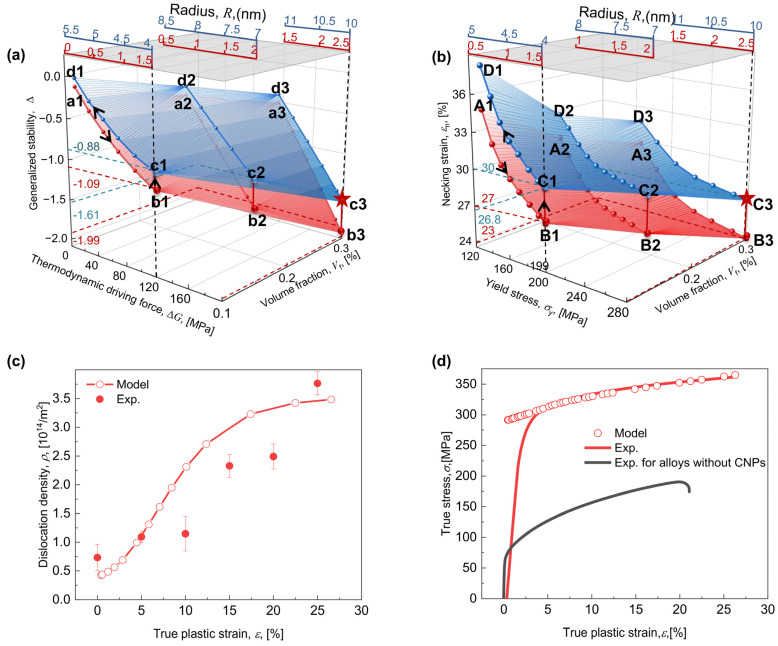
Evolution of (**a**) the thermodynamic driving force ∆G and the generalized stability GS or dislocation, and (**b**) the yield stress $\sigma_{y}$ and the necking strain $\varepsilon_{n}$ with the volume fraction $V_{f}$ and the radius R of CNPs. Evolution of (**c**) the model-predicted and the experimentally measured dislocation density ρ and (**d**) the true stress with the true plastic strain ε for an optimized combination of $V_{f}$ = 0.3% and R = 10 nm. The axes in (**a**,**b**) represent the radius values selected for calculation due to the shearing-dominated mechanism (red) and due to the Orowan-dominated mechanism (blue), corresponding to $V_{f}$ values of 0.1%, 0.2% and 0.3%. The five-pointed stars in figures (**a**,**b**) represent optimization points.

By changing the combinations of $V_{f}$ and R of CNPs, the ∆G-GS and the $\sigma_{y}$-$\varepsilon_{n}$ trade-offs are anticipated, as shown in Figure 12a and Figure 12b, respectively. For each given $V_{f}$, the increased R causes the trade-offs of ∆G-GS and the $\sigma_{y}$-$\varepsilon_{n}$ to emerge, as reflected by three different scales indicating the transition from coherency to order and then to the Orowan-dominated mechanism; e.g., for $V_{f}$ = 0.1%, an inherent quadrilateral a1b1c1d1 forms (Figure 12a), accompanied by the continuous growth of R, corresponding to an inherent quadrilateral A1B1C1D1, indicating the transition of the strengthening mechanism (Figure 12b). As for the coherency-dominated mechanism, the growth in R gives the increased ∆G and the decreased GS (see the red line indicated by a1b1 in Figure 12a), and in turn, the enhanced $\sigma_{y}$ and declined $\varepsilon_{n}$ (see the red line indicated by A1B1 in Figure 12b); for the order-dominated mechanism, a rise in R leads to comparable ∆G but an enhanced GS (see the red line indicated by b1c1 in Figure 12a), and in turn, comparable $\sigma_{y}$ but an enhanced $\varepsilon_{n}$ (see the red line indicated by B1C1 in Figure 12b); and for the Orowan-dominated mechanism, an increase in R provides reduced ∆G and improved GS (see the blue line indicated by c1d1 in Figure 12a), and in turn, the lower $\sigma_{y}$ and higher $\varepsilon_{n}$ (see the blue line indicated by C1D1 in Figure 12b). Accordingly, the higher R for the Orowan-dominated mechanism provides comparable ∆G but a higher GS, corresponding to comparable $\sigma_{y}$ but a higher $\varepsilon_{n}$; e.g., for $V_{f}$ = 0.1%, as R increases from 1.5 to 4 nm, the ∆G holds almost invariable as 120 MPa, whereas the GS increases from −1.09 to −0.88 (see b1–c1 in Figure 12a); this corresponds analogously invariable $\sigma_{y}$ as 199 MPa and a continuously increased $\varepsilon_{n}$ from 27% to 30% (see B1–C1 in Figure 12b). Above model-predicted $\sigma_{y}$/$\varepsilon_{n}$ are well-proved by the experimental results. For $V_{f}=0.1\%$, as R rises from 3 (Figure S6a,b) to 4 nm (Figure S1a,b), $\sigma_{y}$ remains comparable (194 (Figure 4) and 200 MPa (Figure S6e)), whereas $\varepsilon_{n}$ increases from 27% to 31%.

The higher $V_{f}$ gives the increased ∆G and the decreased GS, corresponding to the increased $\sigma_{y}$ and decreased $\varepsilon_{n}$; e.g., as $V_{f}$ increases from 0.1% to 0.3%, the ∆G gradually increases from 120 to 200 MPa while the GS gradually decreases from −1.09 to −1.99 (see b1 → b3 in Figure 12a), corresponding respectively to an increase in $\sigma_{y}$ from 199 to 280 MPa and a decrease in $\varepsilon_{n}$ from 27% to 23% (see B1 → B3 in Figure 12b). Note that as $V_{f}$ increases, the R values for the transition from coherency to order and from order to Orowan-dominated strengthening increase; e.g., as V increases from 0.1% to 0.3%, the R values for the transition from coherency to order-dominated strengthening increases from 1.5 to 2.5 nm, and the R values for the transition from order to Orowan-dominated strengthening increases from 4 to 10 nm, which seems similar to Figure 3d. Above model-predicted $\sigma_{y}$/$\varepsilon_{n}$ are also well-proved by the experimental results. As $V_{f}$ grows from 0.1% (Figure S6a,b) to 0.3% (Figure S6b,d), $\sigma_{y}$ rises from 200 to 300 MPa, whereas $\varepsilon_{n}$ declines from 27% to 22% (Figure S6e).

For increasing $\sigma_{y}$ and $\varepsilon_{n}$ simultaneously, the high ∆G-GS trade-off scale is required to design $V_{f}$ and R. Firstly, the values of $V_{f}$ = 0.3% seem to be decided by the higher ∆G (Figure 11a), which correspond to the higher $\sigma_{y}$ (Figure 11b). On this basis, the optimized R can be chosen under a specific $V_{f}$ based on the high ∆G-GS trade-off scale, e.g., as $V_{f}$ = 0.3%, the value of R = 10 nm corresponding to the higher GS of −1.61 should be selected, in substitution of the R = 2.5 nm corresponding to the lower GS of −1.99 (Figure 11a). Consequently, R= 10 nm will provide a higher $\varepsilon_{n}$ of 26.8% rather than a lower $\varepsilon_{n}$ of 23% for R = 2.5 nm (Figure 11b). The proposed design strategy can be verified by comparing the experimental results and the model predictions obtained for $V_{f}$ = 0.3% and R = 10 nm; see the TEM results shown in Figure S7, where the statistically obtained values for $V_{f}$ and R of CNPs were tested as 0.3% and 10 nm. As shown in Figure 11c, the calculated ρ increases from ~0.7 × 10^14^ m^−2^ to ~3.7 × 10^14^ m^−2^ as a function of ε, in close agreement with the expected values; see the HEXRD profiles with different true plastic strains shown in Figure S8. The tensile stress-strain curve is shown in Figure 11d, where the estimated findings seem in excellent agreement with the model-predicted $\sigma_{y}$ (~300 MPa) and $\varepsilon_{n}$ (~27.5%). Particularly, as compared to the alloy without CNPs, the $\sigma_{y}$ and the $\varepsilon_{n}$ exhibit increases of about 225 MPa and 5%, respectively.

## 7. Conclusions

(1) Considering dislocation nucleation, gliding, and annihilation, a dislocation-based strain hardening framework is established in terms of irreversible thermodynamics, where R and $V_{f}$ of CNPs are coupled through cooperative ∆G and Q of coherency, order, and Orowan strengthening.
(2) The variations in $\sigma_{y}$ are attributed to the modifications in strengthening mechanism, whereas the variations in $\varepsilon_{n}$ are linked to ρ, and in turn to Q, independent of variations in $V_{f}$, $\dot{\gamma }$, or R.
(3) The trade-off between $\sigma_{y}$ and $\varepsilon_{n}$ is as a consequence of the trade-off between ∆G and $V^{*}$. Based on this, the trade-off between $\sigma_{y}$ and $\varepsilon_{n}$ can be broken by improving the $V^{*}$ through cooperating coherency, order, and Orowan strengthening.
(4) Following the high ∆G-high GS criterion, a new strategy is proposed to optimize mechanical properties: high ∆G for dislocations nucleation controlled by $V_{f}$ improving $\sigma_{y}$, and high GS for dislocations gliding governed by appropriate R accelerating $\varepsilon_{n}$.

From the perspective of thermo-kinetics, the present model can be utilized to design $V_{f}$ and R of CNPs according to the high ∆G—high GS criterion to improve $\sigma_{y}$ and $\varepsilon_{n}$, simultaneously. As generally proved in previous research, the Orowan mechanism, compared to other mechanisms, exhibits a lower trade-off between $\sigma_{y}$ and $\varepsilon_{n}$, which is associated with a dramatic decrease in strain hardening rate at the later stage of PDs. Nevertheless, efforts are herein undertaken to raise the $V^{*}$ through the cooperative strengthening of coherency, order, and Orowan mechanism, so that a higher trade-off between ∆G and GS, and between $\sigma_{y}$ and $\varepsilon_{n},$ prevails for the Orowan-dominated mechanism. Firstly, the result is a predictive capability, which allows one to illustrate potential directions for improving both strength and uniform elongation. Secondly, this analysis provides further possibilities for designing CPN-strengthened microstructures with a combination of enhanced strength and elongation.

As far as the authors are aware, in single-phase Al alloys, the $V_{f}$ of CNPs generally falls between 0.07% and 0.47%, with a R ranging from 1.4 to 10.2 nm. For $R<R_{1}$, the coherency strengthening dominates; for $R_{1}<R<R_{2}$, the order strengthening dominates; and for $R>R_{2}$, the Orowan mechanism dominates. $R_{1}$ varies between 1.2 and 2 nm, while $R_{2}$ varies between 3 and 4 nm. The range mentioned aligns with the $V_{f}$ and R designed in this study. It is necessary to confirm the suitability of the high ∆G-high GS design criterion for other metallic-structured materials by additional verification.

## Figures and Tables

**Table 1:** Nomenclature.

Symbol	Description [Unit]	Symbol	Description (Unit)
α	Strengthening coefficient	Q	Kinetic energy barrier (kJ/mol)
$\alpha^{’}$	Geometrical factor	$Q_{0}^{coh}$	Zero-stress energy barrier for coherency strengthening (kJ/mol)
b	Burgers vector [nm]	$Q_{0}^{ord}$	Zero-stress energy barrier for order strengthening (kJ/mol)
C	Temperature-dependent constant	$Q_{0}^{oro}$	Zero-stress energy barrier for Orowan strengthening (kJ/mol)
∆	Generalized stability	$r_{1}$	Contributions ratio for coherency strengthening
∆G	Thermodynamic driving force [MPa]	$r_{2}$	Contributions ratio for order strengthening
$∆G_{\tau }$	Driving force from applied shear stress [MPa]	$r_{3}$	Contributions ratio for Orowan strengthening
$∆G_{\tau_{r}}$	Driving force from gliding resistance [MPa]	R	Radius of CNPs (nm)
dγ	Shear strain interval [%]	ρ	Dislocation density (m^−2^)
dS/dt	total entropy change rate [kJ/(m^2^·s)]	$\rho_{0}$	Initial dislocation density (m^−2^)
$d_{e}S/dt$	Entropy flux rate between system and surroundings [kJ/(m^2^·s)]	$\rho_{m}$	Mobile dislocation density (m^−2^)
$d_{i}S/dt$	Entropy generation rate [kJ/(m^2^·s)]	σ	True stress (MPa)
dU	Dislocations storage energy [kJ/m^2^]	$\sigma_{0}$	Lattice true stress (MPa)
dW	Mechanical work [kJ/m^2^]	$\sigma_{f}$	Forest dislocation true stress (MPa)
$dW_{an}$	Dissipated energies of dislocation annihilation [kJ/m^2^]	$\sigma_{p}$	Precipitate true stress (MPa)
$dW_{ge}$	Dissipated energies of dislocation generation [kJ/m^2^]	$\sigma_{p}^{coh}$	True stress due to coherency strengthening (MPa)
$dW_{gl}$	Dissipated energies of dislocation glide [kJ/m^2^]	$\sigma_{p}^{oro}$	True stress due to order strengthening (MPa)
E	Elastic energy of dislocations per unit length [kJ/m^2^]	$\sigma_{p}^{ord}$	True stress due to Orowan strengthening (MPa)
Γ	Dislocation line tension [N]	$\sigma_{y}$	Yield true stress (MPa)
γ	Shear strain [%]	∂σ/∂ε	Strain hardening rate (MPa)
$\dot{\gamma }$	Shear strain rate [s^−1^]	τ	Applied shear stress [MPa]
${\dot{\gamma }}_{0}$	Reference strain rate [s^−1^]	$\tau_{0}$	Shear resistance of lattice [MPa]
$G_{m}$	Shear modulus of matrix [MPa]	$\tau_{f}$	Shear stress of forest dislocation [MPa]
$k_{1}$	Dislocation storage coefficients	$\tau_{f0}$	Shear stress of forest dislocation at yielding point [MPa]
$k_{1c}$	constant dislocation storage coefficient	$\tau_{r}$	Average shear resistance [MPa]
$k_{2}$	Kinetic recovery coefficients [m^−1^]	$\tau_{fr}$	Shear resistance of forest dislocation [MPa]
$k_{2c}$	constant kinetic recovery coefficient [m^−1^]	${\hat{\tau }}_{r}^{coh}$	Peak shear resistance for coherency strengthening [MPa]
$k_{B}$	Boltzmann’s constant [J/K]	${\hat{\tau }}_{r}^{ord}$	Peak shear resistance for order strengthening [MPa]
K	Resistive force [N]	${\hat{\tau }}_{r}^{oro}$	Peak shear resistance for Orowan strengthening [MPa]
$\hat{K}$	Peak resistive force [N]	$\tau_{y}$	Applied shear stress at yielding point [MPa]
$l^{s}$	Mean gliding distance [m]	T	Absolute temperature [K]
m	Strain rate sensitivity exponent	v	Dislocation gliding velocity [m/s]
N	Number of dislocation jogs per unit length	$v_{0}$	Atomic vibration frequency [s^−1^]
$N^{coh}$	Number of CNPs for coherency strengthening	$V_{f}$	Volume fraction of CNPs [%]
$N^{ord}$	Number of CNPs for order strengthening		
$N^{oro}$	Number of CNPs for Orowan strengthening		

**Table 2:** Chemical composition of the Al-Mg-Si alloy (wt%).

Si	Mg	Fe	Ti	Zn	Cu	Al
1.0–1.5	0.25–0.6	0.15–0.3	≤0.2	≤0.1	≤0.2	Bal

**Table 3:** Model parameters used to approximate the stress-strain data.

Parameter	Unit	Al-Zn-Mg	Al-Mg-Si
$\sigma_{i}$	MPa	200	181
$\sigma_{\infty }-\sigma_{i}$	MPa	458	223
$1/\tilde{\varepsilon }$	/	3.83	3.81
$\sigma_{\infty }$	MPa	658	404
$\sigma_{0}$	MPa	30	55
$\sigma_{s}$	MPa	483	265
$k_{1c}$	/	1.9 × 10^8^	1.06 × 10^8^
$k_{2c}$	m^−1^	2.50	2.49
$\varepsilon_{n}$ (Model/Exp.)	MPa	23.1%/24.2%	25.7%/26.1%

**Table 4:** Physical parameters for model calculations.

Parameter	Unit	Value	Refs.
Strengthening coefficient for flow stress of forest dislocation, α	/	0.15	[5]
Shear modulus of matrix, $G_{m}$	MPa	25.4 × 10^3^	[5]
Burgers vector, b	nm	0.286	[62]
Poisson’s ratio, ν	Wm^−1^K^−1^	0.33	[5]
Shear modulus of the precipitate, $G_{P}$	MPa	37.2 × 10^3^	[5]
Antiphase boundaries energy per unit area, $\gamma_{APB}$	J m^−2^	0.5	[62]
Linear elastic misfit, ϵ	/	0.0179	[62]
Taylor factor, M	ms^−1^	3.06	[62]
Line tension of dislocation, Γ	N	1.072 × 10^−9^	[62]

## Data Availability

The original contributions presented in the study are included in the article and Supplementary Materials, further inquiries can be directed to the corresponding author.

## Supplementary Materials

The following supporting information can be downloaded at: https://www.mdpi.com/article/10.3390/ma17174197/s1, Figure S1: TEM bright image of the coherent nanoprecipitates (CNPs) and corresponding statistical result for $V_{f}=0.1\%$ and $\bar{R}$ = 4 nm; Figure S2: Flow chart of algorithm to calculate the present dislocation-based strain hardening model; Figure S3: Contribution of various strengthening evolving with the true plastic strain ε for various combinations of $\dot{\gamma }$ and $V_{f}$; Figure S4: Contribution of various strengthening evolving with the ε for various combinations of $\dot{\gamma }$ and R; Figure S5: Synchrotron high-energy X-ray diffraction patterns for $V_{f}$ = 0.1% and $\bar{R}$ = 4 nm; Figure S6: TEM bright images of CNPs, corresponding statistical results, and experimental true stress-strain curves for $V_{f}=0.1\%$ and $\bar{R}$ = 3 nm, and for $V_{f}=0.3\%$ and $\bar{R}$ = 6 nm; Figure S7: TEM bright image of CNPs and corresponding statistical result for $V_{f}=0.3\%$ and $\bar{R}$ = 10 nm; Figure S8: Synchrotron high-energy X-ray diffraction patterns for $V_{f}$ = 0.3% and $\bar{R}$ = 10 nm. References [86,87,88,89,90,91,92] are cited in the Supplementary Materials.
