# Supplementary material for: Effect of Coherent Nanoprecipitate on Strain Hardening of Al Alloys: Breaking through the Strength-Ductility Trade-Off
Source: Materials (Basel). 2024 Aug 24;17(17):4197. doi: 10.3390/ma17174197 (PMC11395813; doi:10.3390/ma17174197)
Supplement: Supplementary file 1 [file materials-17-04197-s001.zip › materials-3116843-supplementary.pdf]

materials

MDPI

Supplementary material

Effect of Coherent Nanoprecipitate on Strain Hardening of Al Alloys: Breaking through the Strength-Ductility Trade-Off

Pan Wu [1], Kexing Song [2] and Feng Liu [1,3,*]

[1] State Key Laboratory of Solidification Processing, Northwestern Polytechnical University, Xi'an 710072, China; wupan@mail.nwpu.edu.cn
[2] Henan Academy of Sciences, Zhengzhou 450046, China
[3] Analytical & Testing Center, Northwestern Polytechnical University, Xi'an 710072, China
* Correspondence: liufeng@nwpu.edu.cn

Includes:

Supplementary note Details of dislocation thermo-kinetic model for individual strengthening.

Fig. S1 TEM bright image of the coherent nanoprecipitates (CNPs) and corresponding statistical result for $V_\mathrm{f} = 0.1\%$ and $\bar{R} = 4$ nm.

Fig. S2 Flow chart of algorithm to calculate the present dislocation-based strain hardening model.

Fig. S3 Contribution of various strengthening evolving with the true plastic strain ε for various combinations of $\dot{\gamma}$ and V_f.

Fig. S4 Contribution of various strengthening evolving with the ε for various combinations of $\dot{\gamma}$ and R.

Fig. S5 Synchrotron high-energy X-ray diffraction patterns for $V_\mathrm{f} = 0.1\%$ and $\bar{R} = 4$ nm.

Fig. S6 TEM bright images of CNPs, corresponding statistical results, and experimental true stress-strain curves for $V_\mathrm{f} = 0.1\%$ and $\bar{R} = 3$ nm, and for $V_\mathrm{f} = 0.3\%$ and $\bar{R} = 6$ nm.

Fig. S7 TEM bright image of CNPs and corresponding statistical result for $V_\mathrm{f} = 0.3\%$ and $\bar{R} = 10$ nm.

Fig. S8 Synchrotron high-energy X-ray diffraction patterns for $V_\mathrm{f} = 0.3\%$ and $\bar{R} = 10$ nm.

References

Supplementary note

Details of individual strengthening and dislocation thermo-kinetics

In a regular array of CNPs, the shear resistance τ_{p} for both the shearing and the Orowan bypassing mechanisms is given by [1-3]:

$$\tau_{\mathrm{p}} = \hat{K}/\mathrm{b}\lambda \tag{S1}$$

with λ as the regular array spacing of CNPs along dislocation [1], R and V_{f} the radius and the volume fraction of CNPs, and b the magnitude of Burgers vector. For the coherency strengthening, the formula for the peak resistive force $\hat{K}$ is as follows [2]: $\hat{K}^{\mathrm{coh}} = 4\mathrm{b}R\epsilon\mathrm{G_m}$, with $\mathrm{G_m}$ as the shear modulus of matrix, $\epsilon = (V_{\mathrm{p}} - V_{\mathrm{m}})/3$ the linear elastic misfit, V_{p} the molar volume of CNPs, and V_{m} the molar volume of matrix [4]. Since the CNPs are sharable for the coherency strengthening, the regular array spacing λ of coherency strengthening represents the center-to-center spacing and can be calculated as [2]: $\lambda^{\mathrm{coh}} = R(2\pi/3V_{\mathrm{f}})^{1/2}/(\hat{K}/2\Gamma)^{1/2}$. By substituting the $\hat{K}^{\mathrm{coh}}$ and the λ^{coh} into Eq.(S1), the shear resistance of coherency strengthening $\tau_{\mathrm{p}}^{\mathrm{coh}}$ becomes [5, 6]:

$$\tau_{\mathrm{p}}^{\mathrm{coh}} = \chi(\mathrm{G_m}\epsilon)^{3/2}\left(\frac{RV_{\mathrm{f}}\mathrm{b}}{\Gamma}\right)^{1/2} \tag{S2}$$

with χ as the constant which varies between 2 and 3 [7].

For the order strengthening, the $\hat{K}$ is related to the antiphase boundaries (APB) energy γ_{APB} by the formula [3]: $\hat{K}^{\mathrm{ord}} = \pi R\gamma_{\mathrm{APB}}/4$. As the dislocation penetrates the CNPs, the λ also manifests the center-to-center spacing. Substituting the expressions of $\hat{K}^{\mathrm{ord}}$ and $\lambda^{\mathrm{ord}} = \lambda^{\mathrm{coh}}$ into Eq.(S1), the shear resistance of order strengthening $\tau_{\mathrm{p}}^{\mathrm{ord}}$ is expressed as [7, 8]:

$$\tau_{\mathrm{p}}^{\mathrm{ord}} = 0.81\frac{\gamma_{\mathrm{APB}}}{2\mathrm{b}}\left(\frac{3\pi V_{\mathrm{f}}}{8}\right)^{1/2} \tag{S3}$$

with 0.81 as the statistics parameter.

For the Orowan strengthening, the $\hat{K}$ related to the line tension is provided by [1]: $\hat{K}^{\mathrm{oro}} = (\mathrm{G_m b^2})/(2\pi\sqrt{1-\nu})\ln((2R)/\mathrm{b})$, with ν as the Poisson's ratio. Since the CNPs are bypassed by the Orowan loops, the λ of Orowan strengthening refers to the edge-to-edge spacing and can be given as [9]: $\lambda^{\mathrm{oro}} = [1.538(V_{\mathrm{f}})^{-1/2} - 1.643]R$. Substituting the $\hat{K}^{\mathrm{oro}}$ and the λ^{oro} into Eq.(S1), the shear resistance of Orowan bypassing strengthening $\tau_{\mathrm{p}}^{\mathrm{oro}}$ becomes [1]:

$$\tau_{\mathrm{p}}^{\mathrm{oro}} = \alpha\frac{\mathrm{G_m b}}{\lambda_{\mathrm{f}}\pi\sqrt{1-\nu}}\ln\left(\frac{2R}{\mathrm{b}}\right) \tag{S4}$$

with $\alpha = 0.4$ as the statistical sampling factor [6].

Physically, the kinetic energy barrier Q for dislocation evolution can be generally expressed as [1, 2]:

$$Q = \Delta F - \Delta W = \int_{V_1}^{V_2}\tilde{K}\,\mathrm{b}\mathrm{d}V - K\mathrm{b}V^{*} \tag{S5}$$

with ΔF as the Helmholtz free energy, ΔW the work, $\tilde{K}$ the resistive force profile of discrete particle against distance, K the resistive force of discrete particle to dislocation motion, V_1 and $V_2$the volume corresponding to two equilibrium positions, and V^{*} the activation volume. Since the value of ΔF is difficult to determine, more attention has been paid to the peak resistive force $\hat{K}$ [1, 2, 10]. Conversing the integral variable, Eq. (S5) can be proposed as:

$$Q = \int_{K}^{\hat{K}}\mathrm{b}V^{*}\,\mathrm{d}\tilde{K} \tag{S6}$$

In this situation, Q can be integrated as:

$$Q = Q_0\left(1 - \left(K/\widehat{K}\right)\right) \quad \text{(S7)}$$

with $Q_0 = \int_0^{\widehat{K}} \mathrm{b}V^* \,\mathrm{d}\widetilde{K}$ as the zero-stress energy barrier. For order and Orowan strengthening, analogous to Ref. [1], the obstacle profile can be simplified to a rectangular box and the maximal V^* is assumed as $2R$. For the order strengthening, $Q_0 = Q_0^{\mathrm{ord}} = 2R\widehat{K}^{\mathrm{ord}}\mathrm{b}$, while for the Orowan strengthening, $Q_0 = Q_0^{\mathrm{oro}} = 2R\widehat{K}^{\mathrm{oro}}\mathrm{b}$. However, for the coherency strengthening, the obstacle profile is an energy-dissipating soft-contact obstacle; thus, a 1/3 power factor is introduced rather than a simple rectangular box [1, 2], i.e., $Q_0 = Q_0^{\mathrm{coh}} = 2R^{1/3}\widehat{K}^{\mathrm{coh}}\mathrm{b}$. Following statistical theory [11], the relationship between shear resistive force and shear resistance can be expressed as: $K/\widehat{K} = (\tau/\hat{\tau}_{\mathrm{r}})^i$, with τ as the applied shear stress and $\hat{\tau}_{\mathrm{r}}$ the peak shear resistance. For the order strengthening, $\tau^{\mathrm{ord}} = \tau_0 + \left(((\dot{\gamma}/\dot{\gamma}_0)^m\tau_{\mathrm{f}})^2 + \left(\tau_{\mathrm{p}}^{\mathrm{ord}}\right)^2\right)^{1/2}$ and $\hat{\tau}_{\mathrm{r}}^{\mathrm{ord}} = \tau_0 + \left(\tau_{\mathrm{fr}}^2 + \left(\tau_{\mathrm{p}}^{\mathrm{ord}}\right)^2\right)^{1/2}$, with τ_0 as the shear resistance of lattice, $\tau_{\mathrm{f}} = \alpha \mathrm{G_m}\mathrm{b}\sqrt{\rho}$ the shear stress of forest dislocation, $\tau_{\mathrm{fr}} = \alpha' \mathrm{G_m}\mathrm{b}\sqrt{\rho_{\mathrm{m}}}$ the shear resistance of forest dislocation, α the strengthening coefficient varying within a wide range of 0.2~0.5 [12], m the strain rate sensitivity exponent, α' the geometrical factor that depends on type and arrangement of the interacting dislocations, and ρ_{m} the mobile dislocation density; for the coherency strengthening, $\tau^{\mathrm{coh}} = \tau_0 + \left(((\dot{\gamma}/\dot{\gamma}_0)^m\tau_{\mathrm{f}})^2 + \left(\tau_{\mathrm{p}}^{\mathrm{coh}}\right)^2\right)^{1/2}$ and $\hat{\tau}_{\mathrm{r}}^{\mathrm{coh}} = \tau_0 + \left(\tau_{\mathrm{fr}}^2 + \left(\tau_{\mathrm{p}}^{\mathrm{coh}}\right)^2\right)^{1/2}$; and for the Orowan strengthening, $\tau^{\mathrm{oro}} = \tau_0 + \left(((\dot{\gamma}/\dot{\gamma}_0)^m\tau_{\mathrm{f}})^2 + \left(\tau_{\mathrm{p}}^{\mathrm{oro}}\right)^2\right)^{1/2}$ and $\hat{\tau}_{\mathrm{r}}^{\mathrm{oro}} = \tau_0 + \left(\tau_{\mathrm{fr}}^2 + \left(\tau_{\mathrm{p}}^{\mathrm{oro}}\right)^2\right)^{1/2}$. Specifically, for the regular array, i= 1, and for the Friedel description of the random ones, i = 2/3 [1, 2, 10], which may be appropriate for some obstacles such as non-interacting impurities and precipitates and seems incompatible with interacting obstacles such as forest dislocations following incomplete random. Comparing the Eq.(S7) with the phenomenological relations of Q, i.e., $Q = Q_0(1 - (\tau/\hat{\tau}_{\mathrm{r}})^{\mathrm{p}})^{\mathrm{q}}$, the physical parameter i in the present model appears to be mathematically equivalent to the adjustable parameter p with q = 1 of the Kocks model [10]. In this case, Eq. (S7) should be used with the proper selection of the value of i.

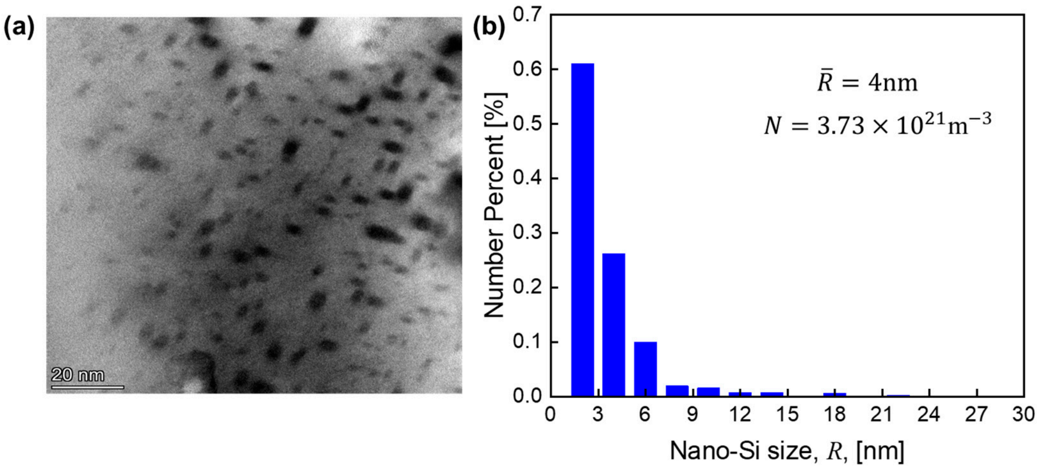

Figure S1. (a) TEM bright image and (b) corresponding statistical results of CNPs for $V_\mathrm{f} = 0.1\%$ and $\bar{R}$ = 4 nm. Note that the number density of particles is obtained by counting the number of CNPs on each image and then dividing by the area of image. Volume fraction of CNPs is calculated by $V_\mathrm{f} = (4/3)\pi N \bar{R}^3$. The results were obtained by measuring and averaging the radius of CNPs using ImageJ software.

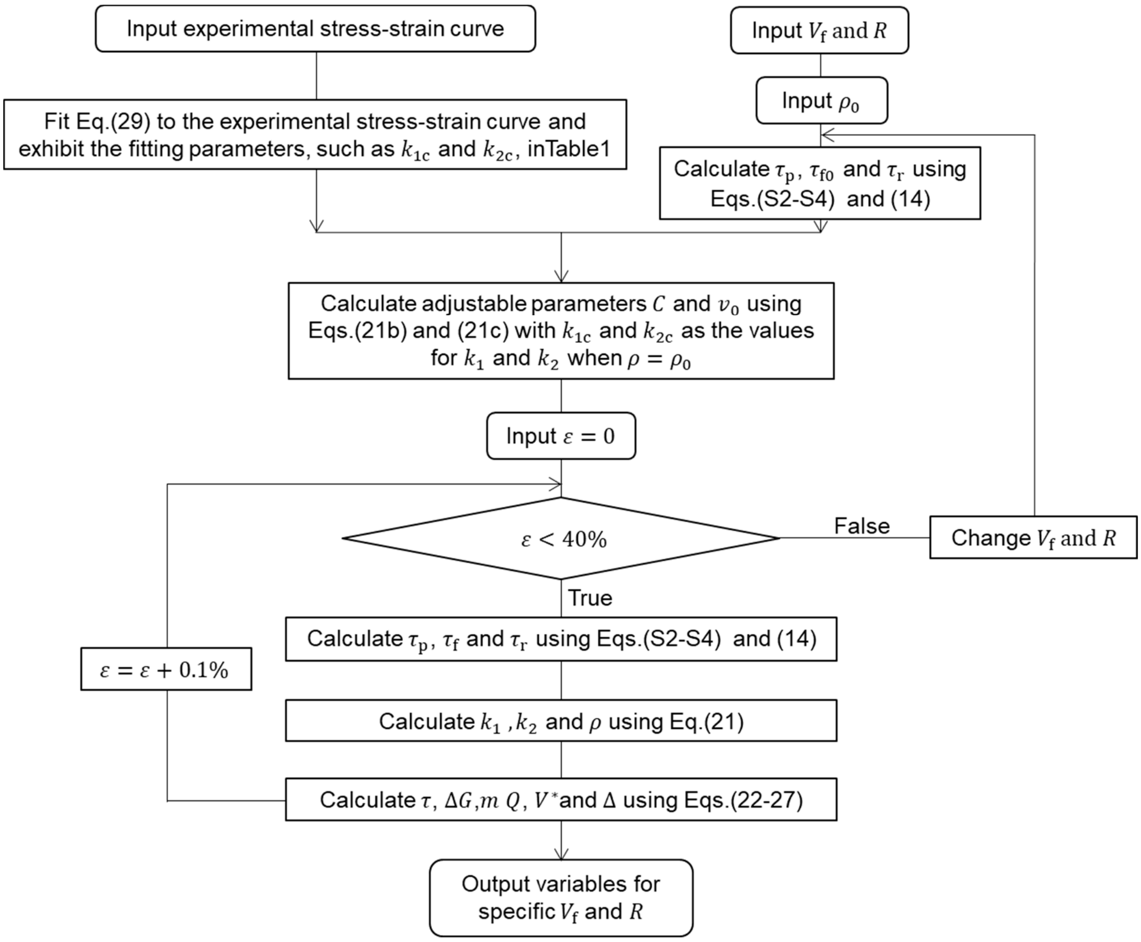

Figure S2. Flow chart of the algorithm to calculate the present dislocation-based strain hardening model.

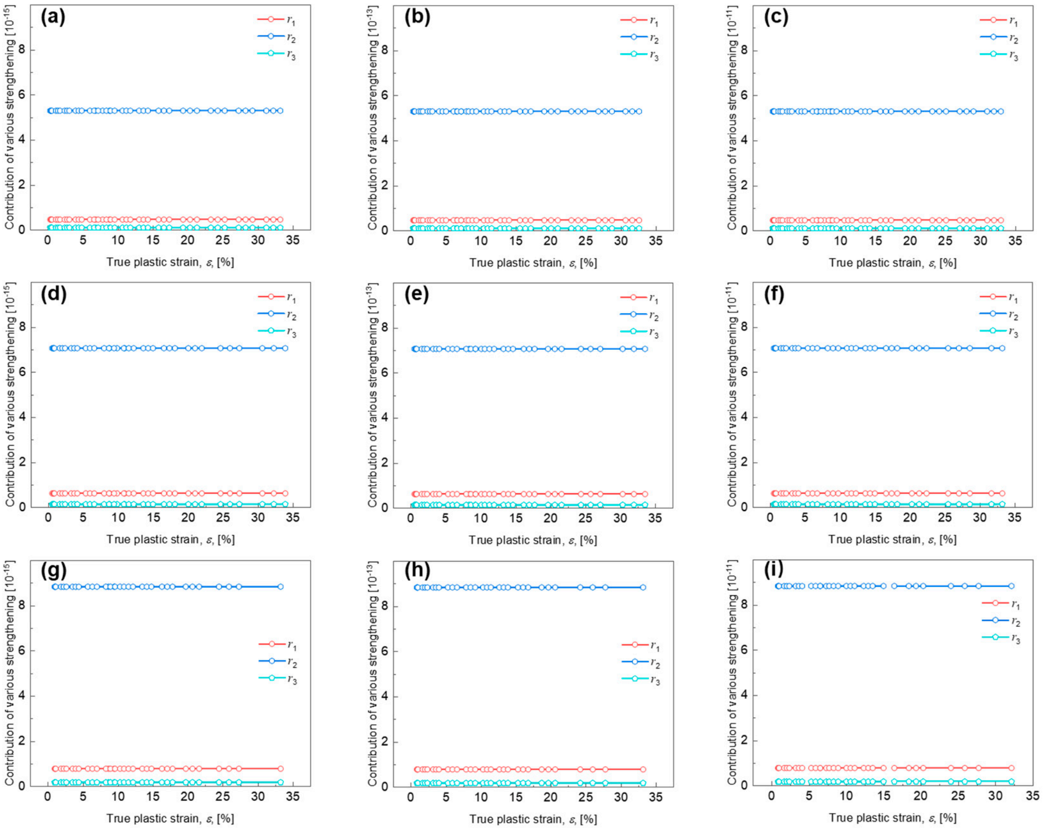

Figure S3. Evolution of the contribution of various strengthening with the true plastic strain ε for various combinations of $\dot{\gamma}$ and V_f with $R = 3$ nm. (a) $\dot{\gamma} = 10^{-4}$ s^{-1} and $V_f = 0.1\%$; (b) $\dot{\gamma} = 10^{-2}$ s^{-1} and $V_f = 0.1\%$; (c) $\dot{\gamma} = 10^{0}$ s^{-1} and $V_f = 0.1\%$; (d) $\dot{\gamma} = 10^{-4}$ s^{-1} and $V_f = 0.2\%$; (e) $\dot{\gamma} = 10^{-2}$ s^{-1} and $V_f = 0.2\%$; (f) $\dot{\gamma} = 10^{0}$ s^{-1} and $V_f = 0.2\%$; (g) $\dot{\gamma} = 10^{-4}$ s^{-1} and $V_f = 0.3\%$; (h) $\dot{\gamma} = 10^{-2}$ s^{-1} and $V_f = 0.3\%$; (i) $\dot{\gamma} = 10^{0}$ s^{-1} and $V_f = 0.3\%$.

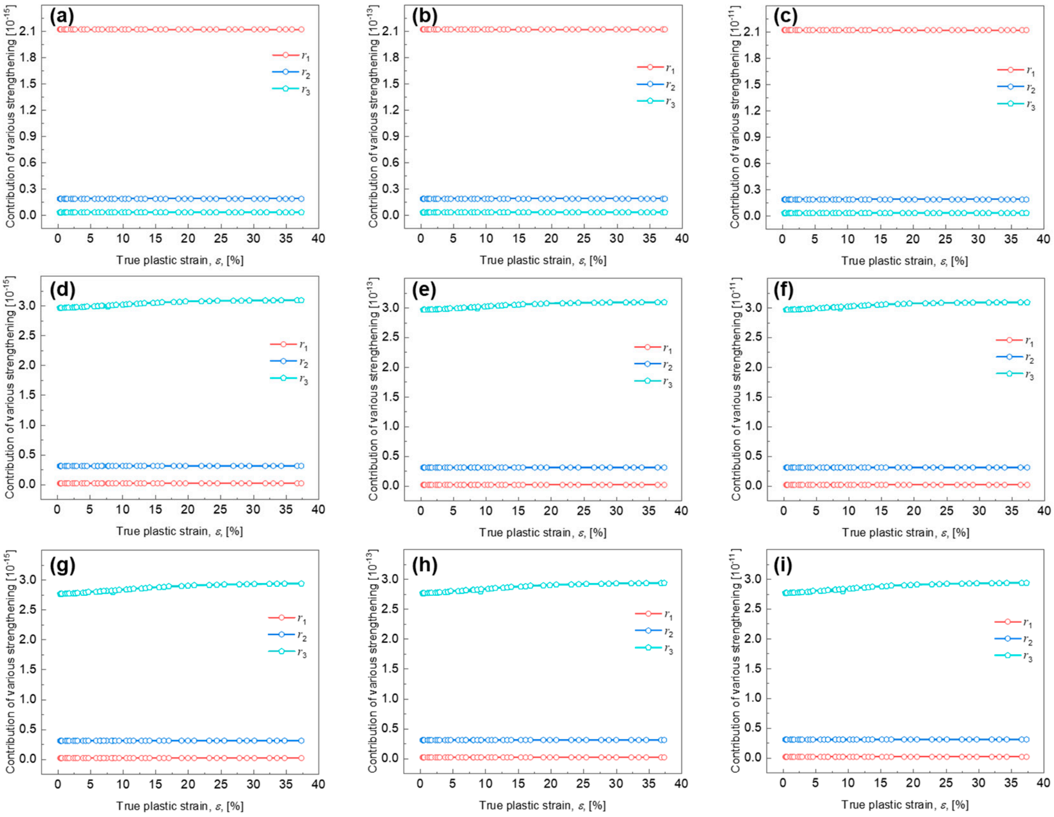

Figure S4. Evolution of the contribution of various strengthening with the true plastic strain ε for various combinations of $\dot{\gamma}$ and R with $V_f = 0.2\%$. (a) $\dot{\gamma} = 10^{-4}$ s^{-1} and $R = 0.7$ nm; (b) $\dot{\gamma} = 10^{-2}$ s^{-1} and $R = 0.7$ nm; (c) $\dot{\gamma} = 10^{0}$ s^{-1} and $R = 0.7$ nm; (d) $\dot{\gamma} = 10^{-4}$ s^{-1} and $R = 7$ nm; (e) $\dot{\gamma} = 10^{-2}$ s^{-1} and $R = 7$ nm; (f) $\dot{\gamma} = 10^{0}$ s^{-1} and $R = 7$ nm; (g) $\dot{\gamma} = 10^{-4}$ s^{-1} and $R = 8$ nm; (h) $\dot{\gamma} = 10^{-2}$ s^{-1} and $R = 8$ nm; (i) $\dot{\gamma} = 10^{0}$ s^{-1} and $R = 8$ nm.

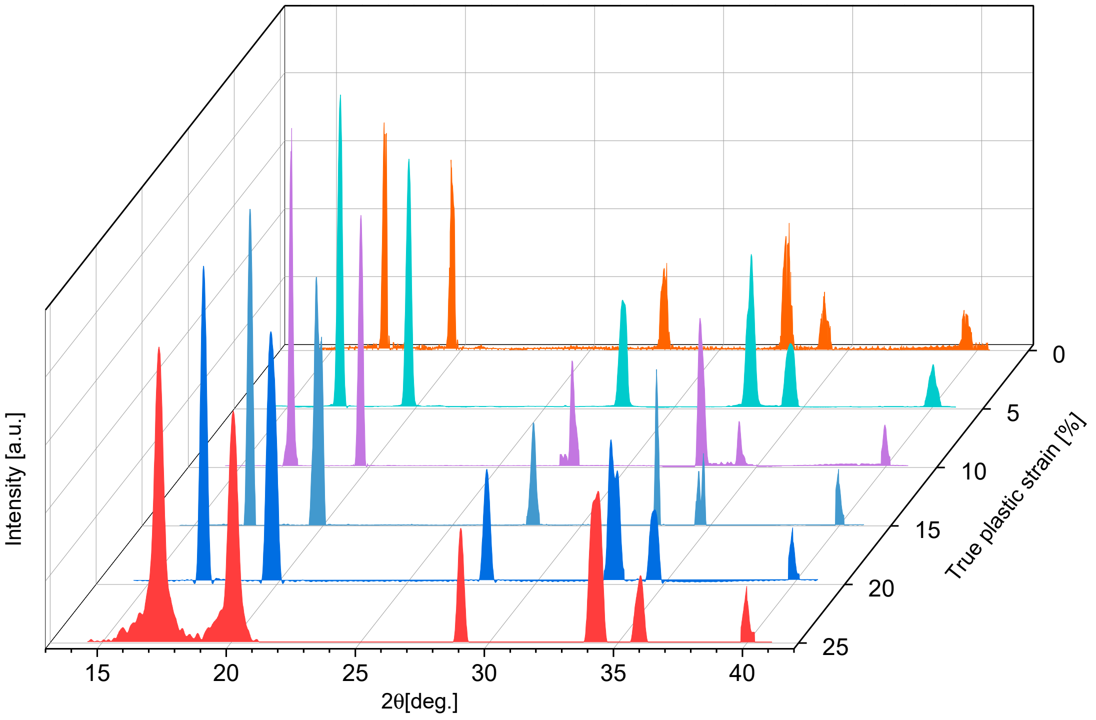

Figure S5. Synchrotron high-energy X-ray diffraction patterns of samples with $\bar{R}$ = 4 nm and V_f = 0.1% of nano-Si deformed at different true plastic strain levels of 0%, 5%, 10%, 15%, 20%, and 25%, respectively.

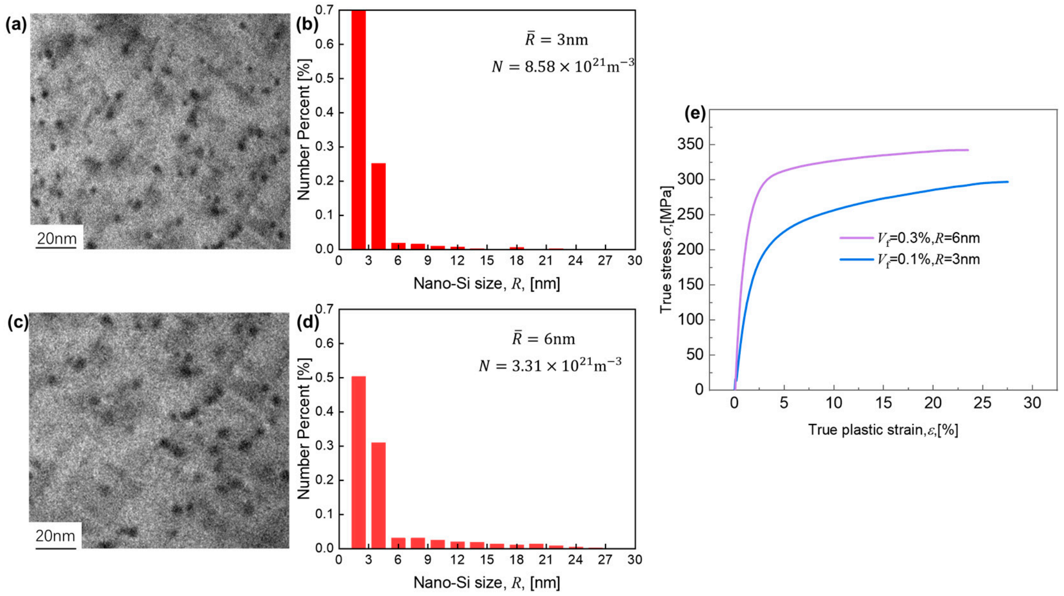

Figure S6. (a) TEM bright image and (b) corresponding statistical result of nano-Si with V_f = 0.1% and $\bar{R}$ = 3 nm. (c) TEM bright image and (d) corresponding statistical result with V_f = 0.3% and $\bar{R}$ = 6 nm. (e) Corresponding experimental true stress-strain curves.

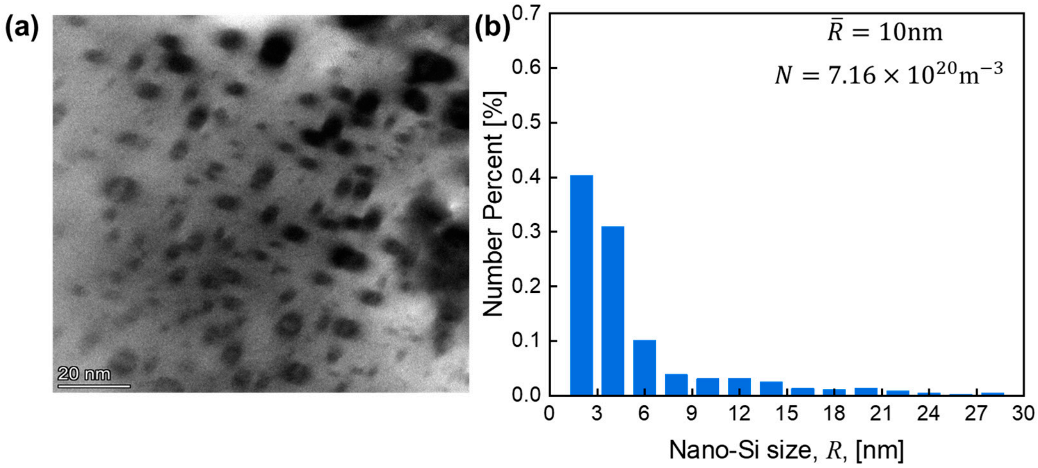

Figure S7. (a) TEM bright image and (b) corresponding statistical result of nano-Si for V_f = 0.3% and $\bar{R}$ = 10 nm.

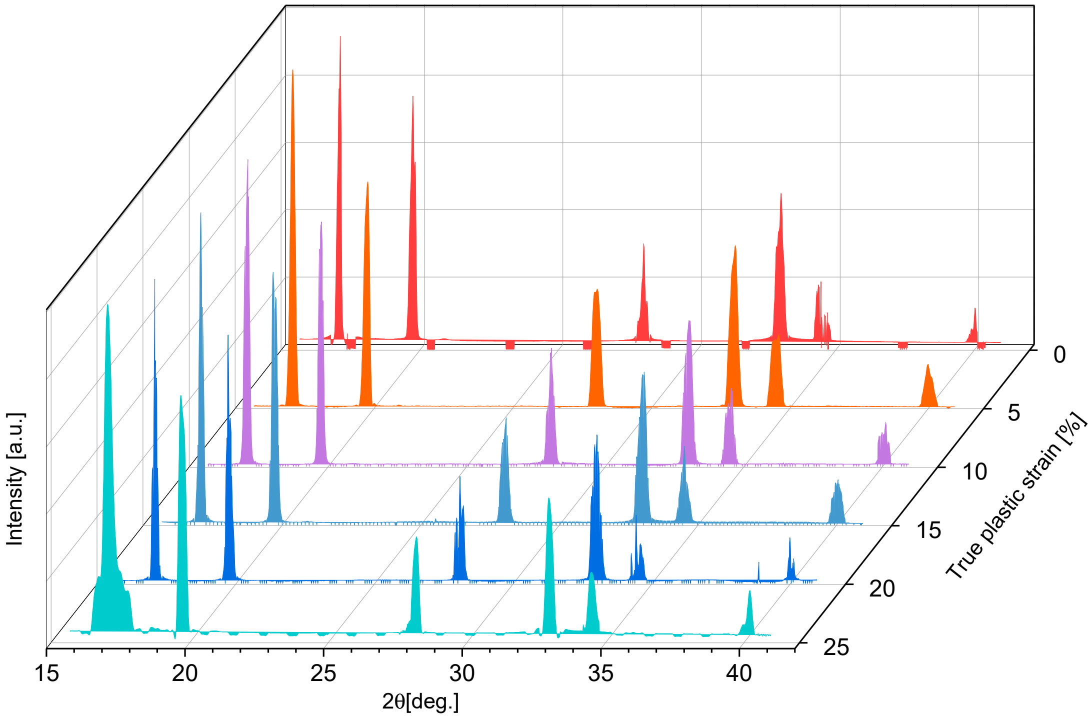

Figure S8. Synchrotron high-energy X-ray diffraction patterns of samples with the combination of V_f = 0.3% and $\bar{R}$ = 10 nm of nano-Si deformed at different true plastic strain levels of 0%, 5%, 10%, 15%, 20%, and 25%, respectively.

References

1. [1] A.S. Argon, Strengthening mechanisms in crystal plasticity, Oxford University Press on Demand, NewYork, 2008.
2. [2] U.F. Kocks, A.S. Argon, M.F. Ashby, Prog. Mater. Sci. 19 (1975) 1-281. https://doi.org/10.1016/0079-6425(75)90007-9
3. [3] A.J. Ardell, Metall. Trans. A 16 (1985) 2131-2165. http://doi.org/10.1007/bf02670416
4. [4] E. Kozeschnik, Momentum Press, New York, 2013.
5. [5] D.A. Luca, D.N. Seidman, D.C. Dunand, Acta Mater. 165 (2019) 1-14. http://doi.org/10.1016/j.actamat.2018.11.031

6. [6] H. Chen, Z. Chen, G. Ji, S.Y. Zhong, H.W. Wang, A. Borbély, Y.B. Ke, Y. Bréchet, Int. J. Plast. 139 (2021) 102971. http://doi.org/10.1016/j.ijplas.2021.102971
7. [7] K.E. Knipling, R.A. Karnesky, C.P. Lee, D.C. Dunand, D.N. Seidman, Acta Mater. 58 (2010) 5184-5195. http://doi.org/10.1016/j.actamat.2010.05.054
8. [8] K. Ma, H. Wen, T. Hu, T.D. Topping, D. Isheim, D.N. Seidman, E.J. Lavernia, J.M. Schoenung, Acta Mater. 62 (2014) 141-155. http://doi.org/10.1016/j.actamat.2013.09.042
9. [9] Y. Zhang, K.Y. Gao, S.P. Wen, H. Huang, Z.R. Nie, D.J. Zhou, J Alloy Compd 610 (2014) 27-34. http://doi.org/10.1016/j.jallcom.2014.04.093
10. [10] Y.Z. Li, M.X. Huang, Int. J. Plast. 138 (2021) 102921. http://doi.org/10.1016/j.ijplas.2020.102921
11. [11] E. Nadgornyi, Prog. Mater. Sci. 31 (1988) 1-530. http://doi.org/10.1016/0079-6425(88)90005-9
12. [12] P. Zhang, K.K. Shi, J.J. Bian, J.Y. Zhang, Y. Peng, G. Liu, A. Deschamps, J. Sun, Acta Mater. 207 (2021) 116682. http://doi.org/10.1016/j.actamat.2021.116682
13. [13] S.J. Andersen, C.D. Marioara, R. Vissers, A. Frøseth, H.W. Zandbergen, Mater. Sci. Eng. A. 444 (2007) 157-169. http://doi.org/10.1016/j.msea.2006.08.084
14. [14] A.K. Gupta, D.J. Lloyd, S.A. Court, Mater. Sci. Eng. A. 316 (2001) 11-17. https://doi.org/10.1016/S0921-5093(01)01247-3
15. [15] P. Schüler, R. Frank, D. Uebel, S.F. Fischer, A. Bührig-Polaczek, C. Fleck, Acta Mater. 109 (2016) 32-45. http://doi.org/10.1016/j.actamat.2016.02.041
16. [16] F. Lasagni, B. Mingler, M. Dumont, H.P. Degischer, Mater. Sci. Eng. A. 480 (2008) 383-391. http://doi.org/10.1016/j.msea.2007.07.008
17. [17] X. Zhang, L.K. Huang, B. Zhang, Y.Z. Chen, F. Liu, Mater. Sci. Eng. A. 794 (2020) 139932. http://doi.org/10.1016/j.msea.2020.139932
